# Hydrogel Implementing Drug Delivery in Cranial Bone Tissue Engineering

**DOI:** 10.3390/jfb17080401

**Published:** 2026-08-14

**Authors:** Martina Salvati, Alessia Vita, Alessandro Arcovito, Ornella Parolini, Federica Tiberio, Wanda Lattanzi

**Affiliations:** 1 Dipartimento di Neuroscienze, Organi di Senso e Torace, Fondazione Policlinico Universitario Agostino Gemelli IRCCS, Largo Agostino Gemelli 8, 00168 Rome, Italy; martina.salvati@unicatt.it; 2 Dipartimento Scienze Della Vita e Sanità Pubblica, Università Cattolica del Sacro Cuore, Largo Francesco Vito 1, 00168 Rome, Italy; alessia.vita@unicatt.it (A.V.); ornella.parolini@unicatt.it (O.P.); 3 Fondazione Policlinico Universitario Agostino Gemelli IRCCS, Largo Agostino Gemelli 8, 00168 Rome, Italy; alessandro.arcovito@unicatt.it; 4 Dipartimento di Scienze Biotecnologiche di Base, Cliniche, Intensivologiche e Perioperatorie, Università Cattolica del Sacro Cuore, Largo Francesco Vito 1, 00168 Rome, Italy; 5 Fondazione IRCCS Casa Sollievo Della Sofferenza, Viale Cappuccini, 71013 San Giovanni Rotondo, Italy

**Keywords:** smart biomaterials, hydrogel-based drug delivery, cranial bone tissue engineering, controlled release, regenerative medicine

## Abstract

Cranial bone defects remain a significant clinical challenge due to their limited intrinsic regenerative capacity and the complexity of coordinating osteogenesis, angiogenesis, and immune responses within a confined and poorly vascularised environment. Conventional approaches, including autologous grafts and synthetic implants, provide structural support but fail to actively modulate the biological processes required for effective bone regeneration. In this context, hydrogel-based systems have emerged as versatile platforms for localized and controlled drug delivery in cranial bone tissue engineering (BTE). This review provides a comprehensive overview of hydrogel-based delivery strategies designed to regulate the spatiotemporal presentation of bioactive agents within cranial defects. The main classes of hydrogels, natural, synthetic, semi-synthetic, and hybrid systems, are discussed in relation to their physicochemical properties and suitability for drug delivery applications. Current delivery approaches are analysed, including cell-free systems (growth factors, peptides, bioactive ions, nucleic acids, and small molecules drugs), cell-based platforms, and multifunctional systems integrating secondary carriers such as nanoparticles (NPs), microparticles (MPs), and extracellular vesicles (EVs). Particular emphasis is placed on how hydrogel design parameters, including crosslinking density, degradation kinetics, and responsiveness to microenvironmental cues, govern therapeutic release and influence regenerative outcomes. Emerging strategies and key translational challenges are also highlighted.

## 1. Introduction

Cranial bone defects represent a major clinical challenge in reconstructive surgery due not only to their structural complexity but also to the difficulty of achieving effective biological regeneration [1,2,3,4,5]. These defects, arising from trauma, tumour resection, or congenital conditions, often require surgical intervention, as critical-sized lesions lack intrinsic healing capacity [6,7,8]. While autologous bone grafting remains the gold standard, its clinical application is limited by donor site morbidity, restricted availability, and unpredictable resorption [9,10,11,12,13]. Alternative approaches, including allografts and synthetic implants (e.g., titanium meshes and PEEK-based substitutes), provide structural support but fail to actively promote osteogenesis, vascularisation, and long-term integration [14,15,16]. In addition, their bioinert nature may impair physiological bone remodelling and increase the risk of complications [3,4,17]. A key limitation underlying current therapeutic strategies is the inability to locally and temporally regulate the biological processes governing bone regeneration. Cranial healing requires a tightly coordinated sequence of events, including inflammation resolution, angiogenesis, and osteogenesis, mediated by multiple bioactive signals [18,19,20,21,22]. However, direct administration of therapeutic agents such as growth factors, small molecules, or extracellular vesicles (EVs) is hindered by rapid degradation, short half-life, and systemic dispersion, leading to poor efficacy and potential off-target effects [23,24,25,26,27,28]. In this context, the development of localised and controlled drug delivery systems has emerged as a central challenge in cranial bone tissue engineering (BTE).

The cranial environment is particularly demanding, being spatially confined, relatively poorly vascularised, and in close proximity to sensitive neural tissues, thus requiring precise spatiotemporal control over therapeutic presentation [19,21]. Hydrogels have gained increasing attention as delivery platforms capable of addressing these limitations [29,30]. Their highly hydrated and tuneable polymer networks enable the encapsulation, protection, and sustained release of bioactive agents directly at the defect site. Importantly, their injectability and conformability allow for minimally invasive administration and adaptation to irregular cranial geometries, making them particularly suitable for localised drug delivery applications [31,32,33,34]. Previous reviews have addressed craniofacial BTE or hydrogel-based scaffolds for bone regeneration, mainly focusing on material composition, scaffold design, physicochemical properties, and osteogenic performance [7,29]. However, a focused synthesis of hydrogels as drug delivery platforms for cranial defects, linking hydrogel design, therapeutic cargo, and release kinetics to the coordinated regulation of inflammation, angiogenesis, and osteogenesis, remains limited.

**Figure 1:**
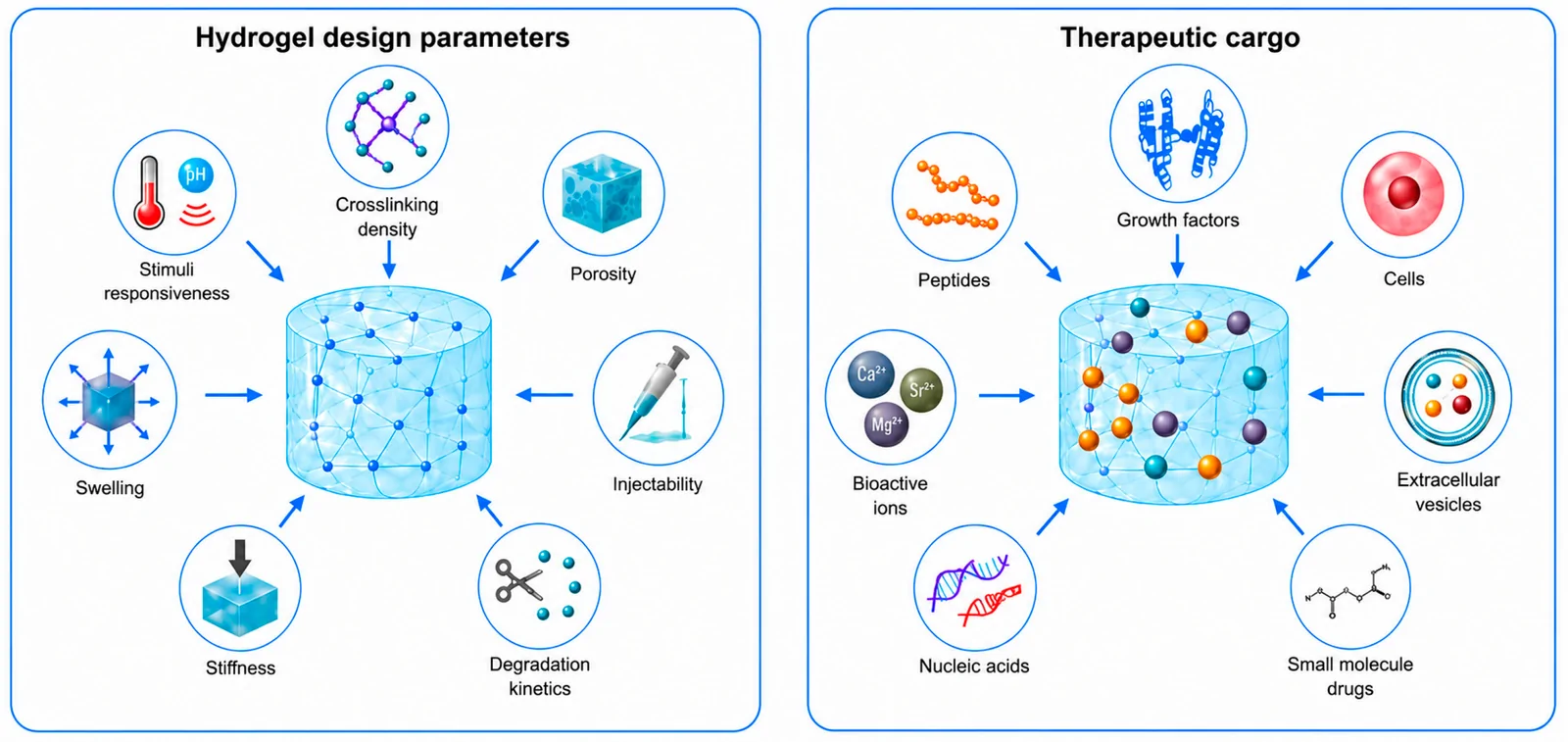
Hydrogel design parameters and therapeutic cargoes in cranial BTE. The left panel summarizes the principal hydrogel properties that can be engineered to regulate local therapeutic delivery, including crosslinking density, porosity, injectability, degradation kinetics, stiffness, swelling behaviour, and responsiveness to internal or external stimuli. The right panel shows the main therapeutic cargoes incorporated into hydrogel systems, including growth factors, peptides, bioactive ions, nucleic acids, cells, extracellular vesicles and small molecule drugs. The interaction between material design and cargo selection determines therapeutic retention, release kinetics, and biological activity within cranial defects.

This review focuses specifically on hydrogel-based materials for drug delivery in cranial BTE, highlighting how their design is leveraged to control the spatial and temporal presentation of therapeutic agents. The discussion is structured around the main delivery strategies, including cell-free systems, cell-based platforms, and multifunctional systems integrating secondary carriers, with particular emphasis on their mechanistic rationale and preclinical performance (Figure 1).

## 2. Types of Hydrogels Used in Cranial BTE

Hydrogels are three-dimensional (3D) crosslinked polymeric networks widely employed in cranial BTE due to their biocompatibility and structural similarity to the extracellular matrix (ECM) [29,30]. In the context of drug delivery, however, their relevance lies primarily in their ability to act as tuneable carriers for localised therapeutic administration [35,36,37].

Hydrogels can be broadly classified into natural, synthetic, and semi-synthetic (hybrid) systems based on their polymer origin. Natural hydrogels, such as collagen, gelatin, alginate, chitosan, and hyaluronic acid, provide intrinsic bioactivity and support cell–matrix interactions but often suffer from limited mechanical stability and rapid degradation [38,39,40]. Synthetic hydrogels, including polyethylene glycol (PEG) and polyvinyl alcohol (PVA), offer highly controllable physicochemical properties and reproducible network architecture, making them particularly suitable for engineering drug release profiles, although they require functionalization to enhance biological interactions [19,41,42]. Semi-synthetic and hybrid systems, such as gelatin methacrylate (GelMA) and methacrylated hyaluronic acid, combine the advantages of both classes, enabling precise control over crosslinking, degradation, and mechanical properties while retaining biological functionality [19,28,43]. A summary of the main hydrogel types, their properties, and typical applications in cranial BTE is provided in Table 1.

These material characteristics provide the foundation for the design of hydrogel-based delivery systems, enabling the development of platforms tailored to the specific biological and pharmacological requirements of cranial bone regeneration.

## 3. Hydrogels for Drug Delivery in Cranial Bone Regeneration

In cranial bone regeneration, hydrogels are not merely passive scaffolds but programmable platforms for localised and controlled therapeutic delivery [35,44,45,46,47]. Their polymeric networks enable the encapsulation and sustained release of bioactive molecules, protecting them from rapid degradation and maintaining effective local concentrations [28,43,48]. The physicochemical properties of hydrogels, including crosslinking density, porosity, swelling behaviour, and degradation kinetics, directly regulate drug diffusion and release profiles [35,44,49]. Injectable and in situ gelling systems further enhance therapeutic efficacy by improving retention at the defect site and minimizing systemic exposure [31,32,33,34,50,51,52]. Importantly, hydrogel-based delivery systems can be engineered to respond to specific microenvironmental cues, such as pH, enzymatic activity, or external stimuli, enabling dynamic and on-demand therapeutic release [53]. This feature is particularly relevant in cranial defects, where inflammation, hypoxia, and limited vascularisation require tightly controlled spatiotemporal modulation of biological signals [19,21].

Based on the nature of the therapeutic payload and delivery strategy, hydrogel-based systems in cranial BTE can be broadly categorized into three main approaches: (i) cell-free delivery systems, (ii) cell-based platforms, and (iii) multifunctional systems integrating secondary carriers.

The following sections examine these strategies in detail, focusing on their mechanistic rationale, design principles, and reported preclinical performance in cranial defect models.

### 3.1. Cell-Free Delivery Strategies

Cell-free (acellular) hydrogel platforms enable the localised delivery of bioactive cues, such as growth factors, cytokines, peptides, small molecules, or nucleic-acid therapeutics, to orchestrate regeneration without exogenous cell transplantation [54,55]. In cranial BTE, these systems are designed to provide spatiotemporally controlled signalling within the defect niche, thereby shaping the early inflammatory response, promoting angiogenesis, and supporting osteogenic commitment and ECM remodelling [56,57]. Because they leverage endogenous repair mechanisms and avoid cell-handling constraints, cell-free hydrogels are frequently discussed as translationally attractive alternatives to cell-based strategies, with optimization shifting toward dose, presentation kinetics, and the coordination of multiple local cues [54,58]. The main classes of therapeutic cargoes employed in hydrogel-based cranial BTE, together with their biological functions, advantages, and limitations, are summarized in Table 2.

#### 3.1.1. Hydrogel Delivering Growth Factors

In the context of bone repair, hydrogels can retain growth factors through different mechanisms, including physical encapsulation, electrostatic interactions, affinity binding, and covalent immobilization (tethering), thereby prolonging their presence at the defect site and enabling spatiotemporally regulated signalling [116]. This is crucial in non-load-bearing environments such as cranial defect models, where hydrogels can be implanted without bearing significant mechanical loads. By contrast, systemic or locally injected free growth factors suffer from rapid degradation, short half-life, and uncontrolled diffusion, which limit their therapeutic efficacy and increase the risk of side effects, including ectopic mineralization and inflammatory responses [59].

Osteogenic growth factors, particularly members of the bone morphogenetic protein (BMP) family, delivered via hydrogels, have consistently shown the ability to trigger robust bone regeneration in cranial defect models [60,61]. Their sequestration within negatively charged hydrogel networks allows for sustained signalling and enhanced interaction with target cells. Moreover, growth factors such as vascular endothelial growth factor (VEGF), platelet-derived growth factor (PDGF), fibroblast growth factors (FGFs), and epidermal growth factor (EGF) contribute to the establishment of a vascularised and well-organized regenerative microenvironment [62,63,117,118]. Beyond directly inducing osteoblast differentiation, these molecules also promote vascularisation within the defect niche, ensuring nutrient delivery essential for bone maturation [119,120].

Covalent tethering has emerged as an attractive approach to further improve the spatial presentation and biological activity of growth factors. By chemically anchoring proteins to the hydrogel network, this strategy minimizes burst release while preserving localised signalling and promoting sustained receptor activation, thereby more closely mimicking the presentation of growth factors by the native extracellular matrix [121]. For example, covalent immobilization of BMP-2 and BMP-9 within MMP-sensitive PEG-based hydrogels significantly enhanced the osteogenic differentiation of encapsulated mesenchymal stromal cells, leading to increased extracellular matrix deposition and mineralization compared with untethered delivery systems over 12 weeks [122].

Overall, growth factors remain among the most potent biological cues for cranial bone regeneration because of their ability to directly regulate osteogenesis and angiogenesis. However, their clinical translation continues to be limited by short biological half-life, rapid diffusion, dose-dependent adverse effects, and high production costs. Consequently, future hydrogel systems should not only prolong growth factor retention but also provide spatially and temporally controlled presentation, thereby reducing the amount of recombinant proteins required while preserving therapeutic efficacy.

##### Bone Morphogenetic Protein

BMPs are members of the transforming growth factor beta (TGF-β) superfamily and represent some of the most potent osteoinductive growth factors in skeletal regeneration. Among them, BMP-2 is the most extensively characterised and is FDA-approved for selected orthopedic indications. Its ability to drive mesenchymal stem cell differentiation toward the osteoblastic lineage has made it a cornerstone molecule in BTE [119,123,124]. However, clinical use is limited by dose-dependent adverse effects, short half-life, and concerns regarding ectopic bone formation and potential tumour-promoting activity [125].

Hydrogel-based delivery systems have been widely explored to improve BMP-2 localization and release kinetics in cranial defect models [126,127,128]. Encapsulation within chitosan-, PEG-, or alginate-based hydrogels enables sustained local release, enhances osteogenic marker (e.g., *Runt-related transcription factor 2-RUNX2*, *osteopontin-OPN*, and *osteocalcin-BGLAP*) expression levels, and accelerates bone matrix deposition in calvarial defects [129]. Clinical evidence further supports this approach: BMP-2-enriched hydrogels have demonstrated bone regeneration outcomes comparable to iliac crest autografts in craniofacial reconstruction [130].

To reduce the supraphysiological doses of BMP-2 often required to achieve effective bone regeneration, combinatorial delivery approaches have been explored. For example, sequential release of BMP-2 together with the anabolic factor insulin-like growth factor-1 (IGF-1) from MP-loaded hydrogels promoted bone regeneration comparable to conventional BMP-2 treatment while requiring lower growth factor doses, highlighting the therapeutic advantage of temporally coordinated signalling. Regenerative efficacy was assessed by micro-computed tomography, histological examination, and immunohistochemical analyses at 4 and 8 weeks post-implantation [64]. Similarly, composite systems such as poloxamer-based or mineral-reinforced hydrogels have demonstrated improved retention and sustained release compared to collagen sponges, resulting in enhanced bone volume and microarchitecture in preclinical cranial models [131].

Emerging approaches emphasize matrix degradability and spatiotemporal control. MMP-sensitive PEG hydrogels have shown that controlled network remodelling promotes cell infiltration and robust defect bridging when delivering BMP-2, whereas non-degradable matrices limit regenerative outcomes [127]. In addition, advanced stimuli-responsive platforms and co-delivery systems integrating pro-angiogenic cues further enhance regenerative performance in complex cranial environments [128,132].

Beyond BMP-2, other family members such as BMP-9 and BMP-4 have demonstrated osteogenic and immunomodulatory potential in preclinical models, suggesting that BMP-based hydrogel systems remain a versatile and evolving strategy for cranial bone regeneration [39,133].

##### Fibroblast Growth Factor

FGFs constitute a family of signalling proteins with pleiotropic effects on cell proliferation, migration, and differentiation, and play critical roles in tissue morphogenesis and repair [134]. Preclinical studies have demonstrated the efficacy of FGF-2 in craniofacial bone regeneration. Early investigations using gelatin-based hydrogels for sustained local delivery showed significant enhancement of bone formation in critical skull defects, establishing proof-of-concept for FGF-mediated cranial repair [135]. Similarly, collagen-based carriers loaded with FGF-2 promoted substantial bone regeneration in mandibular and calvarial defect models, indicating that even short-term controlled exposure can effectively initiate reparative cascades [136].

Another representative study demonstrated that gelatin hydrogels enabling sustained local release of basic fibroblast growth factor (bFGF) significantly enhanced cranial bone regeneration compared with free bFGF administration, ultimately achieving complete defect closure after 12 weeks. Histological and bone mineral density analyses confirmed the superior regenerative efficacy of controlled bFGF delivery, emphasizing the importance of hydrogel-mediated release for maximizing therapeutic outcomes [135].

Recent approaches have combined FGF-2 with other osteoinductive factors. In a calvarial defect model in aged mice, layer-by-layer coatings on a biomimetic calcium phosphate barrier were designed to sequentially release FGF-2 followed by BMP-2 [137,138]. This strategy resulted in a substantial enhancement of bone volume fraction and defect fill, particularly in the central region, when compared with BMP-2 alone. These data suggest that low-dose FGF-2 can potentiate BMP-2-driven repair processes in compromised cranial bone [139].

In a rat calvarial model, an atelocollagen sponge impregnated with recombinant human FGF-2 (rhFGF-2) was applied for vertical guided bone regeneration [140]. This produced significantly greater height and volume of augmented bone beyond the original skeletal contour than control treatments. Histological analyses confirmed the presence of well-vascularised, mature bone tissue within the augmented region, indicating that rhFGF-2 can effectively drive vertical bone gain in a challenging cranial setting [140].

Furthermore, in radiation-induced calvarial defects, FGF-2 maintained pro-angiogenic and osteogenic activity when delivered with hyaluronan gel or biphasic calcium phosphate blocks, although its efficacy could be modulated by adjunctive interventions such as hyperbaric oxygen therapy [141,142]. Overall, these findings show that FGF-2 makes a substantial contribution to cranial bone repair when combined with appropriately engineered collagen-, gelatin-, or hydrogel-based carriers. In this context, FGF-based systems are increasingly considered complementary to BMPs, with promise in boosting angiogenesis, accelerating early bone formation, and improving outcomes in compromised cranial sites.

#### 3.1.2. Hydrogel Delivering Peptides

Functional peptides have emerged as a significant class of bioactive molecules for hydrogel functionalization, complementing growth factor-based strategies [143]. These short sequences recreate key motifs found in ECM proteins (e.g., cell-binding domains), as well as antimicrobial, osteogenic, and immunomodulatory cues. In bone regeneration, peptides exert direct osteogenic effects while also modulating the local immune microenvironment by influencing macrophage polarization toward M1 and M2 phenotypes [65]. This balance is critical, since M1 macrophages dominate the early inflammatory phase and contribute to infection control, whereas M2 macrophages promote tissue repair and remodelling [66,67]. Despite their therapeutic potential, peptides are limited by rapid enzymatic degradation, physicochemical instability, and short in vivo half-life [66]. Hydrogels therefore serve as valuable protective and delivery matrices capable of stabilising peptides and prolonging their local bioactivity.

Self-assembling peptide hydrogels were first reported by Zhang and colleagues in the early 1990s [144]. Through covalent or non-covalent interactions, peptide molecules self-assemble into 3D networks and can be combined with natural or synthetic polymers to generate composite hydrogels [90,145,146,147]. These materials exhibit good biocompatibility and degrade into metabolizable amino acids.

For BTE, these systems must combine biocompatibility, controlled degradation kinetics, adequate mechanical support, and osteoinductive activity, while accommodating the anatomical complexity and mechanical heterogeneity of bone defects [148,149]. Accordingly, peptide sequence design and network architecture can be engineered to tune stiffness, drug-loading, degradability, and bioactivity [150,151].

In cranial defect, peptide-based hydrogels can serve as temporary three-dimensional scaffolds supporting cell infiltration, mineral deposition, and osteoconduction [152,153].

Tsukamoto et al. [154] demonstrated that a self-assembled peptide hydrogel (SPG-178-Gel) increased bone volume 2.5-fold in rat calvarial defects after three weeks. Similarly, Misawa et al. [155] reported enhanced bone regeneration using the self-assembling peptide hydrogel PuraMatrix (RADA16-I) in rat calvarial defects.

Cell-based strategies further enhance regenerative outcomes. Direct stem cell injection leads to low retention and viability [156,157], whereas peptide hydrogels improve localization and protection. Quan et al. [158] showed that peptide hydrogels loaded with rMSCs nearly filled critical-sized cranial defects, while acellular hydrogels induced only marginal bone formation. Kamichika et al. [159] reported superior regeneration using hiPSC-loaded peptide hydrogels compared with controls.

Peptide hydrogels are also effective growth factor carriers. Controlled delivery of BMPs, VEGF, and PDGF enhances bone repair, and sustained-release systems can be achieved via direct loading or incorporation of microspheres [160]. When used for BMP-2 delivery, peptide hydrogels allow for dose reduction and the mitigation of adverse effects [161].

Lee et al. [162] developed a heparin-binding peptide amphiphile system that slowed BMP-2 release and enhanced bone regeneration at reduced doses. In subsequent work, Lee et al. [163] achieved a 100% spinal fusion rate with lower BMP-2 doses and observed a 42% fusion rate even without exogenous BMP-2, suggesting recruitment of endogenous growth factors.

Compared with growth factors, peptides exhibit improved stability and lower production costs, although their osteoinductive potency is generally lower, requiring optimized delivery platforms.

##### Arginine–Glycine–Aspartic Acid

Arginine–Glycine–Aspartic Acid (RGD) is the prototypical integrin-binding peptide widely used to improve cell adhesion on biomaterials. Covalent immobilization of RGD on alginate scaffolds markedly enhances bone formation. However, excessively high ligand concentrations, while augmenting adhesion, can impede cell migration, making optimization of adhesive density essential to balance adhesion and motility [119]. RGD exhibits strong affinity for integrins expressed on osteoblasts. A recent study further highlighted the potential of RGD functionalization in cranial defect repair. Composite dextran–methacrylate (DEXMA) hydrogels functionalized with RGD peptides and co-delivering 3,4-dihydroxyphenylalanine-modified osteogenic peptide P24 (DOPA-P24) together with manganous-manganic oxide (Mn_3_O_4_) nanozymes were described as multifunctional platforms providing adhesive, osteogenic, and antioxidative cues. In a rat critical-sized calvarial defect model (5 mm diameter), the RGD@DEXMA/DOPA-P24/Mn_3_O_4_ formulation enhanced BMSC adhesion and proliferation via the RGD motif, promoted M1-to-M2 macrophage polarization through Mn_3_O_4_-mediated ROS scavenging, and supported neovascularisation. The combined immunomodulatory and osteogenic actions resulted in significantly accelerated cranial bone regeneration compared with control formulations lacking one or more components, underscoring the value of RGD-grafted multifunctional hydrogels for calvarial repair [68].

##### Cathelicidin

LL-37 is the only cathelicidin identified in humans to date and is derived from the C-terminal domain of the precursor hCAP18 [164]. Although it is primarily known for its broad antimicrobial activity, LL-37 has also emerged as a multifunctional peptide involved in immunomodulation, angiogenesis, and tissue repair [69,70,71,72]. These properties make it particularly attractive for bone regeneration, where infection control, vascularisation, and recruitment of reparative cells are all critical. However, its therapeutic use remains constrained by limited stability, susceptibility to proteolytic degradation, and potential cytotoxic effects at higher concentrations [165].

In cranial bone regeneration, LL-37 has shown promising activity primarily through indirect pro-regenerative mechanisms. In a rat calvarial defect model, Kittaka et al. reported that LL-37 enhanced bone repair by increasing the accumulation of STRO-1-positive mesenchymal stem/progenitor cells and CD34-positive endothelial cells within the defect area, suggesting that its main contribution was to promote stem cell recruitment and neovascularisation rather than directly inducing osteogenic differentiation [166], LL-37 was later shown to act synergistically with *BMP-2*-modified MSCs in inflammatory calvarial osteolytic defects, where the combined treatment markedly enhanced both bone formation and vascularisation while also suppressing osteoclastogenesis and bacterial burden [150]. More recently, the regenerative potential of LL-37 was further supported in a rat calvarial defect model using a poly(sebacoyl diglyceride) (PSeD) gel loaded with human adipose-derived stem cells. In this setting, LL-37 promoted osteogenic differentiation of hADSCs, reduced post-implantation inflammation, and significantly accelerated defect healing, highlighting its relevance also in hydrogel-based cranial repair strategies [167].

##### Osteogenic Growth Peptide

Osteogenic growth peptide (OGP) is a conserved tetradecapeptide [168,169] with strong effects on osteoblast maturation and matrix mineralization. When incorporated into gelatin-based hydrogels, OGP enhances osteogenesis by upregulating *BMP-2*, *BGLAP*, and *OPN* expression and promoting calcium phosphate deposition by murine MC3T3-E1 pre-osteoblasts [73]. These findings indicate that OGP-functionalized hydrogels support both early osteogenic commitment and subsequent matrix mineralization, making OGP an attractive candidate for cranial bone regeneration strategies. More recently, temporally functional hydrogel systems have further expanded the therapeutic potential of OGP. Lv et al. developed a silk fibroin-based composite hydrogel incorporating OGP-loaded microgels to achieve sustained peptide release and sequential angiogenic and osteogenic stimulation. The system significantly enhanced BMSC osteogenic differentiation and endothelial cell angiogenic activity in vitro, while in a rat critical-sized calvarial defect model it increased bone volume to approximately 52% after 8 weeks, together with enhanced vascularization and osteogenic marker expression. These findings highlight the therapeutic advantage of temporally coordinated peptide delivery for cranial bone regeneration [74].

##### Copine-7-Derived and Cementum-Related Peptides

Emerging evidence highlights Copine-7 (CPNE7)-derived and cementum-associated peptides as a promising class of biomineralization-regulating molecules with high potential in craniofacial bone regeneration. CPNE7 is a Ca^2+^-dependent phospholipid-binding protein involved in membrane trafficking and ECM secretion, processes closely linked to mineral deposition [75]. In mineralizing tissues, CPNE7 contributes to the regulation of osteogenic differentiation through calcium-dependent signalling and modulation of ECM organization.

To improve stability and bioavailability, bioactive peptides derived from the 321–360 region of CPNE7 have been developed. Among these, the cell-penetrating peptide CPD4 (VNPKYKQKRR) has been shown to promote the expression of osteogenic markers, including *RUNX2* and *BGLAP*, likely through enhancement of intracellular Ca^2+^ signalling and matrix vesicle-mediated mineral nucleation. In a mouse calvarial defect model, CPD4 delivery within collagen-based scaffolds significantly enhanced mineralized tissue formation, suggesting a primary role in regulating cell recruitment, differentiation, and matrix maturation rather than direct mineral nucleation [170].

Cementum-derived proteins such as Cementum Attachment Protein (CAP, also known as HACD1) and Cementum Protein 1 (CEMP-1) play a more direct role in mineral phase initiation. CAP promotes hydroxyapatite nucleation and guides anisotropic crystal growth along collagen fibrils, contributing to the structural organization of mineralized tissues [171,172]. CEMP-1, in addition to regulating mesenchymal cell migration, proliferation, and differentiation, directly facilitates mineral formation by inducing octacalcium phosphate nucleation, a precursor phase of hydroxyapatite crystallization [173,174].

Although originally identified in periodontal tissues, these peptides exhibit a strong capacity to orchestrate both cellular responses and physicochemical aspects of mineralization. Their dual functionality, combining cell-instructive signalling with control over calcium phosphate nucleation, makes them highly attractive for the design of advanced biomaterials for craniofacial bone regeneration.

#### 3.1.3. Hydrogel Delivering Small Molecule Drugs

Small molecule drugs have emerged as an attractive class of therapeutic agents for BTE owing to their relatively low molecular weight, chemical stability, ease of synthesis, and lower manufacturing costs compared with recombinant proteins and growth factors [76]. Many of these compounds exhibit osteogenic, angiogenic, anti-inflammatory, or immunomodulatory properties that can effectively regulate the complex biological processes underlying bone regeneration. However, despite their therapeutic potential, the clinical translation of small molecules is often limited by rapid systemic clearance, short biological half-life, poor local retention, and off-target effects following systemic or local administration. Hydrogel-based delivery systems provide an effective strategy to overcome these limitations by enabling sustained and localised release while maintaining therapeutic drug concentrations within the defect site and minimizing systemic exposure.

Among the most extensively investigated osteoinductive small molecules is simvastatin, a cholesterol-lowering agent that has been shown to stimulate osteoblast differentiation through activation of the BMP-2 signalling pathway while simultaneously promoting angiogenesis via increased VEGF expression [33]. Hydrogel-mediated delivery of simvastatin has consistently demonstrated improved bone regeneration compared with administration of the free drug by prolonging its local retention and reducing the burst release typically associated with conventional formulations [77]. Similarly, metformin, originally developed as an anti-diabetic drug, has attracted increasing attention because of its ability to enhance osteogenic differentiation, modulate inflammatory responses, and activate AMPK-dependent signalling pathways involved in bone formation. Incorporation of metformin into biomaterial-based delivery platforms has been shown to improve bone regeneration while reducing the limitations associated with its rapid diffusion from the implantation site [78,79,80].

Naturally derived bioactive compounds have also emerged as promising candidates for hydrogel-mediated delivery. Icariin, a flavonoid isolated from Epimedium species, has been widely investigated for its capacity to stimulate osteogenic differentiation, enhance angiogenesis, and regulate osteoimmune responses [81]. Recent studies have demonstrated that controlled release of icariin from multifunctional hydrogels or nanoparticle (NP)-assisted delivery systems significantly enhances bone regeneration in critical-sized cranial defects by providing prolonged local bioactivity and synergistically modulating both osteogenesis and the inflammatory microenvironment over 7 days [82,83,84,85]. Other small molecules have also demonstrated promising therapeutic potential when incorporated into hydrogel-based delivery systems. Dexamethasone (DEX), a potent glucocorticoid widely used to induce osteogenic differentiation, has recently been incorporated into chitosan hydrogels through NP-mediated delivery. This strategy enabled sustained drug release while simultaneously promoting macrophage polarization toward the pro-regenerative M2 phenotype and enhancing osteogenic differentiation, ultimately improving bone regeneration in a critical-sized bone defect model [86]. Likewise, deferoxamine (DFO), a hypoxia-mimetic small molecule that promotes angiogenesis through stabilization of hypoxia-inducible factor-1α (HIF-1α) and subsequent upregulation of VEGF signalling, has recently been incorporated into GelMA/demineralized bone matrix composite hydrogels for cranial bone regeneration. Sustained DFO delivery significantly enhanced the osteogenic differentiation of mesenchymal cells in vitro and promoted new bone formation in a rat critical-sized calvarial defect model, as demonstrated by increased BMP-2 and collagen type I expression and improved defect healing compared with DFO-free hydrogels over 12 weeks post-surgery [87]. Combination therapies integrating small molecules with osteogenic proteins have also shown considerable promise. Lei et al. developed a thermosensitive hydrogel enabling the sustained co-delivery of alendronate and BMP-2 from distinct release compartments, simultaneously suppressing osteoclastogenesis while promoting osteogenesis. This dual-release platform significantly enhanced bone regeneration in osteoporotic calvarial defects, illustrating the benefit of coordinating anti-resorptive and osteoinductive therapies [88]. More recently, DNA nanostructures have emerged as efficient carriers to improve the local delivery of osteoinductive small molecules. Huang et al. incorporated asiatic acid-loaded DNA tetrahedra into a HAMA hydrogel, significantly enhancing drug retention and controlled release while simultaneously promoting angiogenesis, suppressing osteoclastogenesis, and stimulating osteogenesis. Mechanistically, the system reprogrammed mitochondrial oxidative phosphorylation through STAT3 signalling, ultimately improving vascularised calvarial bone regeneration [89].

Owing to their broad spectrum of biological activities and favourable physicochemical properties, small molecule drugs are increasingly being combined with multifunctional hydrogel platforms capable of providing spatially and temporally controlled release. This strategy not only enhances local therapeutic efficacy but also enables the simultaneous modulation of osteogenesis, angiogenesis, and the inflammatory microenvironment, making small molecules valuable components of next-generation hydrogel-based systems for cranial bone regeneration.

Compared with recombinant proteins, small molecule therapeutics generally offer greater physicochemical stability, lower production costs, and easier manufacturing. Nevertheless, their relatively lower biological potency and rapid diffusion from the defect site require advanced hydrogel formulations capable of maintaining sustained local concentrations. Future multifunctional hydrogel platforms combining small molecules with complementary therapeutic cargos may further improve the coordinated regulation of osteogenesis, angiogenesis, and the inflammatory microenvironment.

#### 3.1.4. Hydrogel Delivering Bioactive Ions

Bioactive ions have emerged as key regulators of bone regeneration and are increasingly incorporated into hydrogel platforms as cost-effective and stable alternatives to growth factors. When embedded within hydrophilic polymer networks, ions such as calcium (Ca^2+^) [91], zinc (Zn^2+^) [92], magnesium (Mg^2+^) [90], copper (Cu^2+^) [93], and silicon (Si^4+^) [94] can be released in a localised and sustained manner, modulating osteogenesis, angiogenesis, and immune responses.

Within the bone microenvironment, Ca^2+^ regulates osteoblast differentiation through activation of calcium-sensing receptors and stimulation of BMP-2 signalling [95]. Zn^2+^ functions as a cofactor for enzymes involved in mineralization and ECM remodelling [96], whereas Mg^2+^ enhances osteoblast viability and differentiation via PI3K/Akt pathway activation [97]. Cu^2+^ and Si^4+^ further contribute to collagen maturation and angiogenic signalling, supporting vascularised bone formation [98]. Because these ions exhibit narrow therapeutic windows, controlled and sustained release is essential to avoid cytotoxicity or aberrant mineralization. In this context, hydrogels provide a tuneable matrix capable of regulating ionic diffusion, maintaining local concentrations, and synchronizing release with scaffold degradation [48].

In cranial bone regeneration, ion-releasing hydrogel systems are increasingly engineered to address the complex interplay between inflammation, angiogenesis, and osteogenesis. Recent studies have further highlighted the importance of temporally coordinated ion delivery to regulate the osteoimmune microenvironment. Jin et al. developed a temporal immunomodulatory hydrogel incorporating zinc/calcium phosphate hybrid NPs capable of sequentially releasing Ca^2+^ and Zn^2+^ ions. This strategy promoted early M1 macrophage activation to facilitate infection control, followed by M2 polarization to support osteogenesis, ultimately enhancing angiogenesis and cranial bone regeneration in a rat model of infected cranial defects, noted by the significantly reduced bacterial load in granulation tissue harvested from the defect site after 7 days [99]. Thermo-responsive PCLA–PEG–PCLA hydrogels incorporating strontium (Sr)- and Ca^2+^- based ceramics have demonstrated injectability, sustained drug and ion release, and significant enhancement of bone regeneration in rat calvarial defects [33,100]. Similarly, dynamic self-healing alginate–chitosan hydrogels containing Ca^2+^, nanohydroxyapatite, and magnesium oxide (MgO) have shown strong tissue adhesion, antibacterial activity, and upregulation of osteogenic and angiogenic markers in irregular cranial defects [101]. Magnesium ion-mediated regulation of the osteoimmune microenvironment has recently been further explored using hybrid hydrogel systems. Yang et al. developed an alginate-based Mg^2+^-releasing hydrogel incorporated into a TPMS titanium scaffold, which recruited a CCN3^+^ mesenchymal stem cell population, activated Wnt/β-catenin signalling, and promoted macrophage polarization toward the M2 phenotype, significantly enhancing critical-sized calvarial bone regeneration [175]. Moreover, magnesium-based immunomodulatory hydrogels have recently been shown to regulate both innate and adaptive immune responses during bone repair. Ni et al. developed a thermosensitive HA/HPCH hydrogel loaded with nano-MgO that promoted M2 macrophage polarization together with T-cell activation, thereby enhancing angiogenesis, osteogenic differentiation, and in situ cranial bone regeneration without requiring exogenous cells or osteogenic factors [176].

Additional formulation strategies have been developed to further refine ionic delivery within hydrogel matrices. For example, gelatin-based cryogels incorporating strontium-enriched silica reservoirs enabled prolonged local ion release, enhancing osteogenic gene expression and improving craniofacial defect repair in osteoporotic models [177]. Likewise, GelMA-based systems functionalized through Ca^2+^ chelation demonstrated immunomodulatory effects, promoting macrophage polarization toward a pro-regenerative phenotype while supporting vascular ingrowth and bone formation in calvarial defects [48]. Multifunctional ion-releasing hydrogels have also been adapted for osteoporotic bone regeneration. Qin et al. developed an injectable phosphocreatine-grafted hydrogel incorporating teriparatide-coated Sr/Zn phosphate-functionalized Zn–Cu particles, providing sustained release of osteogenic ions together with anabolic signalling. Besides enhancing angiogenesis and osteogenesis, the system inhibited osteoclast activity and bacterial growth, resulting in significantly improved repair of osteoporotic calvarial defects [178].

Collectively, these studies highlight ion-releasing hydrogels as multifunctional platforms capable of modulating the biochemical microenvironment of cranial defects. By combining structural support with controlled ionic signalling, these systems offer a promising strategy to orchestrate immune regulation, angiogenesis, and osteogenesis in a coordinated and spatially confined manner.

#### 3.1.5. Hydrogel Delivering Nucleic Acids

Nucleic acid-based molecules are increasingly incorporated into hydrogel systems to achieve precise molecular control over osteogenesis, angiogenesis, and immune modulation in cranial bone repair. By providing a protective 3D matrix, hydrogels enhance nucleic acid stability, improve cellular uptake, and enable sustained and localised gene regulation within critical-sized defects.

Among programmable DNA-based constructs, aptamers and tetrahedral framework nucleic acids (tFNAs) have gained attention due to their structural stability and high specificity. DNA–gelatin double-network hydrogels functionalized with BMSC-targeting aptamers have demonstrated enhanced endogenous stem cell recruitment and robust calvarial bone regeneration [102]. Similarly, thermogelling DNA-based matrices incorporating tFNAs and angiogenic aptamers have simultaneously promoted osteogenesis and endothelial activation, improving cranial defect repair [103]. More advanced multifunctional constructs combining aptamers, *miR-21*, and BMP-2 within tFNA frameworks and embedded in injectable chitosan hydrogels further enhanced cell homing, matrix mineralization, and new bone formation in critical-sized models [104,105]. Collectively, these systems illustrate the potential of programmable DNA–hydrogel platforms for coordinated in situ regeneration.

Gene-silencing approaches based on siRNA and anti-microRNA (miRNA) delivery have also been integrated into hydrogel matrices to modulate endogenous inhibitory pathways. Co-delivery platforms incorporating BMP agonists and noggin-targeting siRNA achieved simultaneous activation of BMP signalling and suppression of its antagonist, significantly enhancing osteogenic differentiation and calvarial bone formation [106]. Sulfonated chitosan hydrogels functionalized with noggin-siRNA similarly enabled sustained release and improved osteogenesis [107]. Sequential systems combining early MSC recruitment (stromal cell-derived factor-1α, SDF-1α) with sustained *antimiR-138* delivery further demonstrated enhanced cranial regeneration [108]. Beyond plasmid DNA and siRNA, messenger RNA delivery has recently emerged as a promising strategy for localised bone regeneration. Li et al. developed a hybrid extracellular vesicle–GelMA hydrogel system for sustained delivery of VEGFA mRNA, combining the protective properties of EVs with the controlled release characteristics of GelMA. This platform significantly enhanced angiogenesis, osteogenic differentiation, and vascularised bone regeneration in rat calvarial defects, demonstrating the translational potential of hydrogel-mediated mRNA therapy [109].

miRNAs, potent post-transcriptional regulators of bone formation, represent another major class of hydrogel-delivered nucleic acids [110]. Controlled release of *miR-200c*-encoding plasmids from gelatin-coated scaffolds accelerated calvarial repair [111], while GelMA-based hydrogels delivering sticky-end-modified tFNA–*miR-29c* complexes enhanced Wnt signalling and bone regeneration [112]. Similarly, thermosensitive chitosan-based hydrogels incorporating *miR-26a* nanoparticles improved bone regeneration, vascularization, and mechanical properties in a rat calvarial defect model. Critical-sized defects treated with this complex showed significantly increased bone volume and mineral density at 8 weeks, together with enhanced blood vessel formation and improved mechanical properties of the regenerated tissue [113]. Finally, plasmid DNA delivery through hydrogel matrices enables localised and sustained gene expression. Cryoprinted alginate hydrogels carrying BMP-2 plasmid polyplexes preserved plasmid activity and significantly increased osteogenic marker expression and bone volume in critical-sized calvarial defects. At 8 weeks post-implantation, histomorphometric and micro-CT evaluations showed that plasmid BMP-2-based scaffolds significantly enhanced new bone formation, resulting in up to a 46% increase in bone volume compared with controls [114].

By enabling spatially confined, temporally controlled modulation of gene expression, hydrogel-mediated nucleic acid delivery systems provide a powerful alternative to protein-based therapies and support coordinated regulation of osteogenesis, angiogenesis, and immune responses within the defect microenvironment.

### 3.2. Hydrogels as Cell-Based Delivery Systems

Hydrogels represent highly versatile platforms for cell-based cranial bone regeneration owing to their hydrated 3D architecture, injectability, and tuneable biochemical and mechanical properties. In BTE, cell-based strategies predominantly rely on mesenchymal stem cells (MSCs) derived from various sources, including bone marrow-derived MSCs (BMSCs) and adipose-derived stem cells (ADSCs), which are integrated within supportive biomaterial matrices [115,179]. Representative cell-based hydrogel strategies for cranial bone regeneration are summarized in Table 3.

Beyond their differentiation potential, transplanted stem cells actively regulate the local immune microenvironment. Their regenerative efficacy has been closely associated with the ability to modulate macrophage polarization and attenuate excessive inflammation [180,181]. Macrophages are central mediators of biomaterial-induced immune responses [182] and exist along a functional spectrum ranging from pro-inflammatory M1 to pro-regenerative M2 phenotypes [183]. By promoting an M2-skewed immune response, stem cell-based approaches can mitigate inflammation and enhance tissue regeneration, providing a strong rationale for the development of cell-laden hydrogel systems for cranial repair.

Within this framework, hydrogels act as injectable, defect-conforming carriers capable of encapsulating cells, preserving their viability, and presenting them within a biomimetic ECM-like environment. However, a major limitation remains the low engraftment efficiency and functional survival of transplanted cells, as a significant proportion undergo early apoptosis due to insufficient support for adhesion, proliferation, and mechanotransduction. To address these challenges, biomaterial systems have been extensively engineered to enhance cell survival, regulate cell–matrix interactions, and direct lineage-specific differentiation [37,184,185,186,187]. Beyond direct cell transplantation, biomimetic acellular systems have recently been designed to reproduce the regenerative functions of native bone marrow. Che et al. engineered a bone marrow-mimetic hydrogel incorporating cell-free fat extract, which simultaneously promoted haemostasis, macrophage polarization toward an anti-inflammatory phenotype, endothelial network formation, and osteogenic differentiation, resulting in highly vascularised bone regeneration in critical-sized calvarial defects [188].

Collectively, the available evidence demonstrates that MSC-loaded hydrogels provide robust regenerative outcomes by combining the structural support of biomaterials with the paracrine and differentiation potential of transplanted cells. Nevertheless, issues related to cell source variability, manufacturing complexity, storage, regulatory approval, and cost continue to limit their clinical translation. These challenges have stimulated increasing interest in acellular alternatives capable of reproducing the therapeutic effects of MSCs while improving standardization and scalability.

**Table 3:** Cell-based hydrogel delivery systems in cranial BTE. Summary of the principal cell populations incorporated into hydrogel systems for cranial bone regeneration, highlighting their biological functions, advantages, limitations, and representative applications.

Cell Source	Representative Examples	Main Biological Effects	Advantages	Main Limitations	Reference
BMSCs	GelMA, collagen, alginate hydrogels	Osteogenesis, immunomodulation	Extensive preclinical evidence	Limited survival, donor variability	[189,190,191,192,193,194,195,196,197,198,199]
WJ-MSCs	PCL/GO scaffolds, PLA/CNT scaffolds	Osteogenesis, immunomodulation, angiogenesis	Non-invasive harvest, high proliferation, low immunogenicity	Limited hydrogel evidence	[200,201,202,203]
ADSCs	GelMA, µRB hydrogels, fibrin hydrogels	Angiogenesis, osteogenesis	Abundant, easy harvest	Variable osteogenic commitment	[35,128,204,205,206,207,208,209,210,211,212]
Alternative MSCs	OE-MSCs, DPSCs	Osteogenic differentiation	Craniofacial tissue relevance	Limited validation	[213,214]
Osteoblast-like cells	HBO cells + PEG-MAL/JAG1	Matrix mineralization	Direct osteogenic commitment	Limited applicability	[215]

#### 3.2.1. MSC-Based Strategies

MSCs represent the most extensively investigated cell source for cranial BTE owing to their osteogenic differentiation potential, trophic activity, and immunomodulatory functions [216]. Depending on their tissue of origin, MSC-based hydrogel strategies can be broadly categorized into bone marrow-derived, adipose-derived, and alternative craniofacial stem cell populations. Cell-based systems have demonstrated superior regenerative performance in many preclinical studies but remain substantially more challenging to translate clinically than acellular formulations owing to regulatory and manufacturing constraints.

##### Bone Marrow-Derived MSCs

BMSCs remain the gold-standard cell population for hydrogel-assisted cranial bone regeneration due to their robust osteogenic commitment and extensive preclinical validation in calvarial defect models. To overcome diffusion limitations associated with bulk hydrogels and improve adaptability to irregular cranial defects, hydrogel microspheres (HMs) have emerged as injectable building blocks capable of assembling into microporous scaffolds that support cell adhesion, migration, and proliferation [217,218]. For instance, GelMA-based microspheres encapsulating BMSCs significantly enhanced bone formation in rat critical-sized calvarial defects compared with acellular controls, highlighting the essential contribution of transplanted stem cells to the regenerative outcome [189]. Advanced biomimetic strategies have combined BMSCs with immune cells to recreate a regenerative bone niche. Deng et al. developed microfluidically generated, photocrosslinked silk fibroin methacryloyl (SilMA) microspheres co-encapsulating BMSCs, RAW264.7 macrophages, catalase, and oxygen-releasing CaO_2_–hydroxyapatite particles [190]. The inorganic component provided sustained oxygen release for up to 30 days, while catalase reduced hydrogen peroxide-associated oxidative stress. In vitro, live/dead, CCK-8, ALP, Alizarin Red S, gene-expression, and transcriptomic analyses showed sustained cell viability during 28-day culture, enhanced osteogenic mineralization, increased angiogenic signalling, and macrophage polarization toward the M2 phenotype. In critical-sized mouse calvarial defects, micro-CT at 4 and 8 weeks demonstrated the highest BMD, BV/TV, and trabecular number in the complete microsphere formulation, while histology and OCN, OPN, RUNX2, CD31, and CD206 staining confirmed mature bone formation, vascularization, and immunomodulation. Nevertheless, the system remains limited by its multicomponent fabrication, validation in a small-animal model, and the absence of long-term degradation and large-animal data [190].

Co-delivery strategies, combining MSCs with osteogenic and angiogenic cues, have also shown synergistic osteoinductive and immunomodulatory effects. In this context, photo-crosslinked GelMA hydrogels incorporating bioactive glass and BMSCs (BG/BMSCs@GelMA) have been shown to enhance osteogenic differentiation in vitro and to promote angiogenesis, bone regeneration, and M2 macrophage polarization in critical-sized calvarial defects [191]. Similarly, injectable alginate/collagen/calcium sulfate (CaSO_4_) hydrogels enabled high MSC viability (>90%) and osteogenic differentiation, achieving effective repair of critical-sized calvarial defects in rats [192].

More advanced strategies integrate gene therapy within cell-laden hydrogels. Sun et al. [193] developed an injectable methacrylated gelatin (mGL) hydrogel encapsulating human BMSCs together with recombinant adeno-associated virus encoding BMP-2 (rAAV-BMP-2). Following visible light crosslinking, this system enabled localised gene delivery and sustained BMP-2 expression by the encapsulated cells, inducing osteogenic differentiation without exogenous growth factor supplementation. In a murine critical-sized cranial defect model, this gene-activated hydrogel significantly enhanced bone regeneration, highlighting the potential of combining stem cell delivery with localised gene activation strategies [193]. Gene-engineered hydrogels have also been explored to rejuvenate endogenous stem cell function in aged bone. Zhu et al. developed an “inside-out” hydrogel delivering SIRT3-loaded nanovectors while simultaneously recreating a pro-regenerative extracellular microenvironment through iron-chelating polymer chemistry. This dual intracellular and extracellular strategy restored BMSC function, promoted angiogenesis and osteogenesis, and significantly enhanced bone regeneration in aged rat calvarial defects [194].

Mechanical stimulation has also emerged as a relevant adjunctive strategy. BMSC-laden type I collagen hydrogels exposed to low-intensity pulsed ultrasound enhanced osteoblast-specific marker expression and improved mineralized tissue formation in a critical-sized calvarial defect model in NOD scid gamma (NSG) mice, demonstrating the importance of mechanoresponsive hydrogel–cell systems for cranial regeneration [195]. In parallel, BMSC-laden void-forming hydrogels were fabricated by DLP bioprinting a 15% GelMA/10% dextran emulsion, in which dextran removal generated interconnected pores that improved molecular diffusion and cell migration. Compared with standard GelMA, the porous construct enhanced BMSC proliferation, YAP nuclear localization, ALP activity, and osteogenic gene expression. In 6 mm rat cranial defects, it achieved 67% new bone coverage at 8 weeks, compared with 45% for standard GelMA and 26% for untreated defects, as confirmed by micro-CT, histology, and COL1/OCN staining. However, pore formation reduced the mechanical properties of the hydrogel, indicating that matrix stiffness requires further optimization [196].

Other studies have focused on multi-component regenerative systems. Li et al. [197] proposed a dual-delivery strategy combining demineralized bone matrix (DBM) powder and hypoxia-preconditioned BMSCs within an injectable self-healing hydrogel for rabbit cranial bone defects. While the hydrogel alone was insufficient to heal the defect, the combined hydrogel/DBM/BMSC system achieved the best regenerative outcome, outperforming both cell-only and DBM-only formulations, thereby highlighting the advantage of simultaneously providing osteogenic cells and matrix-derived cues [197]. Scaffold architecture has also been shown to influence the therapeutic efficacy of MSC-based hydrogels. Fang et al. developed a radially aligned nanofibrous scaffold incorporating a GelMA hydrogel encapsulating BMSCs, where the hydrogel preserved cell viability and prolonged paracrine activity while hydroxyapatite provided sustained osteoconductive cues. This coordinated immune–angiogenic–osteogenic regulation markedly improved critical-sized calvarial defect repair [198].

Thermoresponsive injectable systems have also been explored to improve cell handling and orthotopic bone formation. Collagen/poly-γ-glutamic acid (Col/γ-PGA) hydrogels loaded with mBMSCs and BMP-2 displayed low viscosity at room temperature and increased mechanical stiffness at physiological temperature, forming an interconnected porous structure that supported cell proliferation and osteogenic gene expression. In a mouse calvarial critical-sized defect model, this platform promoted effective orthotopic bone formation within only 3–4 weeks [199].

##### Wharton’s Jelly-Derived MSCs

Wharton’s jelly-derived mesenchymal stromal cells (WJ-MSCs) have emerged as a promising alternative MSC source for craniofacial bone regeneration owing to their non-invasive procurement, high proliferative capacity, low immunogenicity, and robust immunomodulatory and paracrine properties [200]. Moreover, WJ-MSCs exhibit strong osteogenic differentiation potential under appropriate biochemical and biophysical stimulation, making them attractive candidates for BTE applications [200].

Although bone marrow-derived MSCs remain the most extensively investigated cell source for hydrogel-assisted bone regeneration, increasing evidence supports the use of WJ-MSCs in craniofacial applications. Most available studies, however, have employed solid bioactive scaffolds rather than injectable hydrogels. In one representative study, electrospun PCL scaffolds were surface-modified with graphene oxide and Cissus quadrangularis extract and seeded with 4 × 10^5^ human WJ-MSCs per scaffold [201]. The constructs were implanted into 8 mm critical-sized calvarial defects in Wistar rats and compared with untreated defects and scaffolds lacking either cells or bioactive components. Bone regeneration was evaluated at 6 and 12 weeks using radiography, micro-CT, bone mineral density measurements, and histology. At 12 weeks, the WJ-MSC/PCL–GO–Cissus construct produced nearly complete defect closure and the highest measured bone mineral density, reaching 310 mg cm^−3^ compared with 100 mg cm^−3^ for the acellular PCL–GO–Cissus scaffold and 29.5 mg cm^−3^ for untreated defects. Histology further showed increased osteoblast and osteocyte density and matrix mineralization without evident inflammation or necrosis. Nevertheless, the differences in bone mineral density were not statistically significant, cell survival and fate were not directly tracked, and the use of a non-injectable electrospun scaffold limits its direct relevance to hydrogel-based delivery [201]. Similarly, PLA/CNT scaffolds supported WJ-MSC-mediated bone formation in 8 mm rat calvarial defects. Oral silymarin further increased new bone formation compared with the scaffold/WJ-MSC construct alone (*p* < 0.05). The effect was further increased by oral silymarin, with significantly greater new bone formation than the scaffold/WJ-MSC construct alone (*p* < 0.05). However, this study used a non-injectable scaffold and relied mainly on histomorphometric assessment [202]. Beyond conventional scaffold-based approaches, engineered WJ-MSC constructs have also demonstrated promising regenerative outcomes. Wang et al. reported that WJ-MSC sheets overexpressing *miR-196a-5p* exhibited enhanced extracellular matrix deposition, osteogenic differentiation, and mineralization, resulting in significantly improved calvarial defect healing compared with unmodified cell sheets [203]. These findings indicate that genetic modulation may further enhance the regenerative potential of WJ-MSCs for craniofacial bone repair.

Overall, WJ-MSCs represent a promising yet still underexplored cell source for cranial BTE. Their favourable biological profile, combined with their high proliferative capacity, immunomodulatory activity, and osteogenic potential, makes them attractive candidates for integration into multifunctional hydrogel platforms. However, despite the growing evidence supporting their regenerative capabilities, studies specifically combining culture-expanded WJ-MSCs with drug-delivering hydrogels for cranial or craniofacial bone regeneration remain scarce, highlighting an important opportunity for future research [200].

##### Adipose-Derived Stem Cells

Adipose-Derived Stem Cells (ADSCs) are also attractive for cranial repair due to their strong proliferative and multilineage differentiation potential [204]. They are abundant, easily harvested, resistant to hypoxia, and secrete high levels of angiogenic factors under low-oxygen conditions [205,206,207]. In a rabbit calvarial defect model, ADSC sheet–endothelial progenitor cell composites promoted both osteogenesis and angiogenesis and supported substantial defect repair and integration [208].

In another study, rat ADSCs were encapsulated at 1 × 10^6^ cells mL^−1^ within a 15% microwave-synthesized GelMA hydrogel, which was injected into 6 mm critical-sized calvarial defects and photocrosslinked in situ for 40 s [209]. The regenerative effect of the construct was evaluated with or without photobiomodulation using polychromatic light at 600–1200 nm every 48 h. After 20 weeks, micro-CT showed that the ADSC-loaded GelMA group achieved 60.62 ± 6.34% mineralized matrix formation without photobiomodulation, increasing to 79.93 ± 3.41% with light treatment (*p* = 0.002). Histology and BrdU immunostaining confirmed new bone formation and the persistence of transplanted ADSCs near the regenerating tissue. However, ADSC-loaded GelMA without photobiomodulation was not significantly superior to acellular GelMA, and residual hydrogel remained detectable at 20 weeks, indicating suboptimal degradation [209]. Injectable gelatin-based microribbon (µRB) hydrogels supported ASC survival (81% vs. 27% in conventional hydrogels) and accelerated mineralized bone repair, demonstrating the critical role of paracrine signalling for regeneration in a critical-sized cranial defect mouse [35,210]. Co-delivery with osteogenic factors such as BMP-2 in µRB hydrogels significantly accelerated bone formation, achieving up to 90% defect refilling by week 8 [211]. In osteoporotic models, Sr-doped mesoporous silica NPs embedded in ASC-laden gelatin cryogels improved osteogenic marker expression and bone regeneration [177]. Combining ASCs with growth factor-loaded hydrogels (e.g., BMP-2 in heparin-conjugated fibrin) enhanced calvarial repair, highlighting the importance of bioactive cues in ASC-based cranial regeneration [128].

ASCs can also be engineered to undergo chondrogenesis prior to implantation, promoting endochondral ossification and superior calvarial healing. For example, BMP-2 expressing ASCs seeded in gelatin scaffolds achieved ~86% defect closure via endochondral ossification, demonstrating scaffold-mediated control of lineage specification in critical-sized calvarial defect model [212].

##### Alternative Craniofacial MSC Sources

Beyond conventional bone marrow and adipose-derived MSCs, craniofacial-relevant cell populations have emerged as alternative candidates for calvarial regeneration because of their developmental origin and accessibility. In one study, human olfactory ectomesenchymal stem cells (OE-MSCs) were encapsulated at 1 × 10^6^ cells in an injectable type I collagen hydrogel optimized at 7 mg mL^−1^ [213]. In vitro, the construct maintained cell viability and proliferation and promoted matrix mineralization and the expression of *RUNX2*, *COL1*, *OPN*, *OCN*, and *ALP* over 21 days. In vivo, the cell-laden hydrogel was implanted in 7 mm critical-sized rat calvarial defects and evaluated at 4 and 8 weeks using cell tracking, micro-CT, and H&E staining. DiI-labelled OE-MSCs remained detectable at the defect site after 72 h, while the cell-laden group achieved approximately 10% bone volume fraction at 8 weeks, significantly exceeding the limited healing observed with the acellular hydrogel and untreated controls. Although these findings support OE-MSCs as an accessible osteogenic cell source, regeneration remained incomplete, and the xenogeneic model, short-term cell tracking, and absence of biomechanical assessment limit conclusions regarding long-term efficacy. Other craniofacial-relevant MSC populations such as dental pulp stem cells (DPSCs) have also shown promise. A composite alginate/Matrigel hydrogel containing bioactive glass MPs promoted the osteogenic differentiation of encapsulated DPSCs by modulating the local microenvironment, supporting its potential use for craniofacial bone regeneration applications [214].

#### 3.2.2. Osteoblast-like Cells

Human bone-derived osteoblast-like (HBO) cells represent a lineage-committed population with potential advantages for craniofacial bone regeneration, particularly in paediatric applications. In one study, paediatric HBO cells were encapsulated within a 4% PEG-4MAL hydrogel containing RGD adhesive peptides and JAGGED1 (JAG1)-presenting Dynabeads, providing localised and sustained presentation of the Notch ligand without exogenous BMP-2. In vitro, JAG1 significantly enhanced HBO mineralization and increased ALPL expression by 3.5-fold at day 7, COL1A1 by 5.1-fold, and BGLAP by 12.3-fold at day 14. The system was subsequently evaluated in 4 mm critical-sized parietal defects in NOD-SCID mice using hydrogels containing 100,000 paediatric HBO cells, with a second transcutaneous dose administered at week 4. At 8 weeks, micro-CT showed that JAG1-containing hydrogels increased regenerated bone volume by approximately 1.5-fold compared with HBO cells alone, while Masson’s trichrome and COL1A1 immunostaining confirmed enhanced collagen-rich bone matrix formation. Mechanistically, JAG1 promoted osteoblast commitment through non-canonical Notch signalling involving p70 S6K phosphorylation. Nevertheless, the need for repeated administration, the use of immunodeficient mice, and the absence of biomechanical evaluation limit the immediate clinical translation of this BMP-free strategy [215].

### 3.3. Hydrogels Integrating Secondary Drug Delivery Systems for Cranial Regeneration

Integration with secondary drug delivery platforms represents an advanced strategy to refine the performance of hydrogel-based systems in cranial regeneration. By incorporating nanoscale or microscale carriers within the hydrogel matrix, it becomes possible to further modulate release kinetics, protect labile bioactive agents, and reduce premature diffusion or degradation. Recent approaches have combined hydrogels with NPs, liposomes, MPs, and EVs to create hierarchical delivery constructs capable of multi-stage or stimulus-responsive release [219,220,221,222]. Because these secondary delivery systems differ substantially in terms of their physicochemical properties, loading capacity, release mechanisms, and biological functions, they are discussed separately in the following subsections, covering NP-based systems (Section 3.3.2), EVs-based systems (Section 3.3.3), and MPs-based systems (Section 3.3.4). The main secondary delivery systems integrated into hydrogels and their respective advantages and limitations are summarized in Table 4.

**Figure 2:**
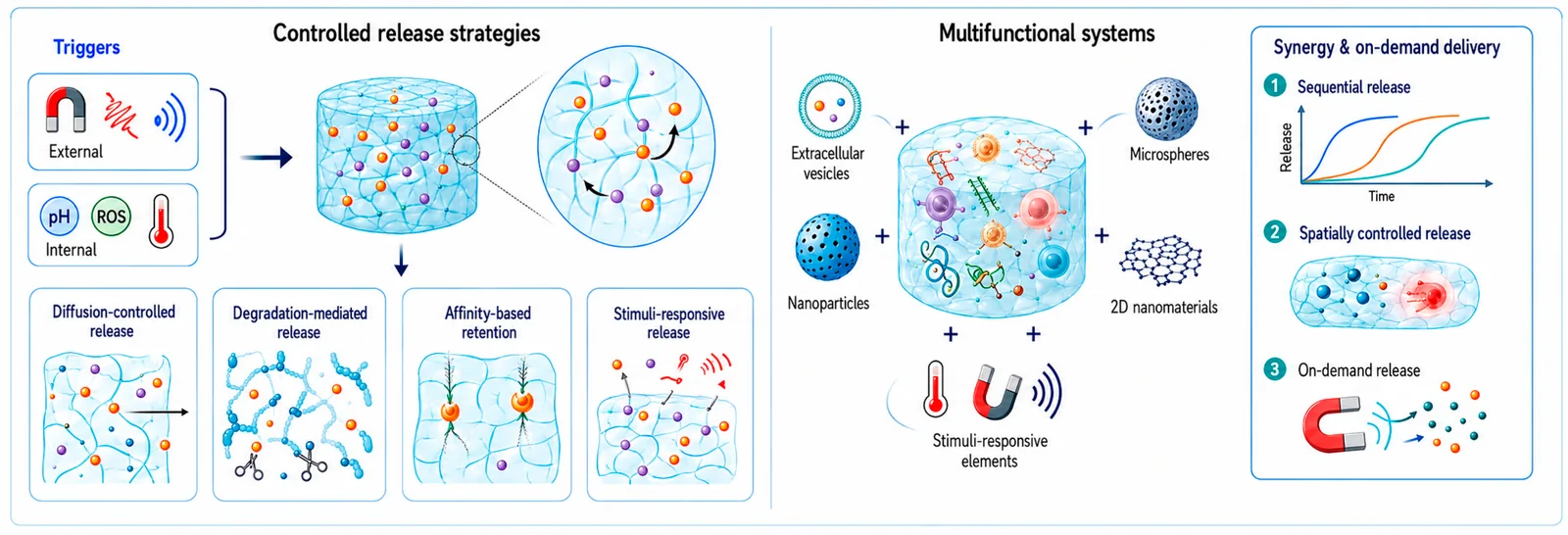
Controlled release strategies and multifunctional hydrogel systems. The left panel provides a visual representation of the principal mechanisms that govern the process of therapeutic release from hydrogel matrices. These mechanisms include diffusion-controlled, degradation-mediated, affinity-based, and stimuli-responsive release. These systems have been observed to respond to endogenous cues, such as pH, reactive oxygen species, enzymes, or temperature, as well as to externally applied stimuli, including magnetic fields and ultrasound. The right panel illustrates multifunctional hydrogels incorporating secondary delivery components, including EVs, NPs, microspheres, two-dimensional nanomaterials, and stimuli-responsive elements. The combination of these elements enables a sequential, spatially controlled, and on-demand delivery mechanism, thereby facilitating the coordinated regulation of immunomodulatory, angiogenic, and osteogenic processes.

In these hybrid systems, the hydrogel primarily provides structural conformity and localised retention, while the embedded carriers introduce an additional level of control over molecular stability and release dynamics [221,252,253]. Rather than functioning as simple reservoirs, such composite platforms enable spatially confined and sequential presentation of therapeutic cues within the defect niche. This layered delivery architecture represents an important step toward improving precision and robustness of bioactive signalling in complex craniofacial environments [254,255]. The main controlled release mechanisms and multifunctional hydrogel strategies discussed in this review are schematically summarized in Figure 2.

#### 3.3.1. Preparation Methods of Composite Hydrogel

The incorporation of secondary drug-delivery systems into hydrogel matrices can be achieved through several engineering strategies that modulate structural organization, release kinetics, and biological performance of the resulting composites. Four principal approaches are commonly described: (i) polymerization of hydrogels in the presence of drug-loaded carriers, (ii) physical incorporation into pre-formed hydrogels, (iii) in situ synthesis of the particulate phase within the hydrogel, and (iv) polymer-coated carrier systems forming core–shell “nanogels” [197,256,257].

Polymerization of hydrogel matrices in drug-loaded carrier suspensions is one of the most widely employed approaches, particularly for NP–hydrogel composites [258,259]. In this method, hydrogel precursors (e.g., acrylamide, PEG methacrylates, or dimethylaminoethyl methacrylate) and crosslinkers are introduced into a suspension of drug-loaded carriers, followed by chemical or photoinitiated polymerization. This strategy enables homogeneous encapsulation and is compatible with a wide range of organic and inorganic particles. However, it may induce particle aggregation during gelation and requires post-polymerization purification to remove unreacted monomers and byproducts, potentially affecting sensitive therapeutics [259].

Physical incorporation into pre-formed hydrogels represents a straightforward and biocompatible alternative. Carriers are introduced after hydrogel formation through swelling, diffusion, mixing, or repeated dehydration–rehydration cycles. Both synthetic hydrogels (e.g., polyacrylamide, PEGDA, PVA) and natural matrices (e.g., collagen, fibrin, hyaluronic acid, chitosan, alginate) have been widely used [260]. Swelling-mediated loading is particularly effective; for example, cyclic deswelling in an organic solvent followed by reswelling in a carrier suspension enables deep penetration of NPs into the network. Although this approach avoids harsh polymerization conditions, it is limited by weaker carrier–matrix interactions and potential carrier leaching, especially at low crosslinking densities [261].

In the in situ synthesis approach, metal or metal-oxide NPs are generated directly within the hydrogel matrix. Charged ions diffuse into the network and are subsequently reduced, chemically, thermally, or photochemically, into nanoscale crystals. This method yields highly dispersed particles with minimal aggregation and strong interactions with the polymer network [262]. However, it is restricted to systems that can be formed via ionic reduction and is not suitable for organic or biomolecular cargoes such as EVs, polymeric NPs, or lipid-based carriers.

The fabrication of carrier-coated hydrogel composites involves the formation of a polymeric shell around a particulate core, producing nanogel or microgel structures capable of encapsulating diverse therapeutic agents [263]. This core–shell architecture provides steric stabilization, reduces aggregation, enhances protection from enzymatic degradation, and enables incorporation of sensitive biomolecules (e.g., enzymes, nucleic acids, hydrophobic drugs) [264]. Techniques such as double-emulsion processing or interfacial polymerization are commonly employed, although they can be technically complex and may leave residual organic solvents requiring careful removal [265].

Overall, the preparation strategy determines the microstructure, mechanical properties, and release behaviour of composite hydrogels. Polymerization ensures uniform distribution but may affect bioactivity, while physical incorporation offers versatility at the expense of retention. In situ synthesis provides high homogeneity for inorganic particles, and core–shell systems enhance molecular protection with increased complexity.

#### 3.3.2. NP-Integrated Hydrogels

NP-integrated hydrogels represent a refined strategy to enhance therapeutic performance of hydrogel-based delivery systems in cranial bone regeneration. By incorporating nanoscale carriers within polymeric networks, these hybrid constructs enable hierarchical control over drug release while simultaneously improving mechanical stability and biofunctional responsiveness [266]. NPs serve as secondary reservoirs capable of encapsulating small molecules, proteins, nucleic acids, or bioactive ions, thereby reducing premature diffusion and mitigating burst release [52,234,258,261,267,268]. Their high surface-to-volume ratio and tuneable surface chemistry allow for the modulation of loading efficiency, release kinetics, and biological interactions [27,66,269]. When embedded within hydrogels, these nanoscale systems provide spatial confinement and protection of therapeutic cargo, while the surrounding matrix ensures localised retention within cranial defects. Moreover, physicochemical properties of NPs, such as magnetic, optical, or pH-responsive behaviour, can be engineered to achieve stimuli-responsive or targeted delivery, offering additional levels of control in complex bone healing environments [27,29,270,271].

Overall, NP-integrated hydrogels provide one of the most versatile platforms for controlled drug delivery because they enable protection of labile therapeutics, sequential release, and multifunctional cargo loading. Organic NPs generally offer greater versatility and biodegradability, whereas inorganic NPs additionally provide intrinsic osteoconductive properties through therapeutic ion release. However, increasing formulation complexity may also complicate large-scale manufacturing and regulatory translation.

##### Inorganic NPs

Inorganic NPs are increasingly incorporated into hydrogel matrices to enhance mechanical properties, bioactivity, and the controlled delivery of therapeutic agents. Materials such as hydroxyapatite (HAp), calcium phosphate (CaP), mesoporous silica NPs (MSNs), bioactive glass, metal–organic frameworks (MOFs), and magnetic NPs provide osteoconductive cues while also acting as secondary reservoirs for drugs and bioactive ions [230].

A variety of multifunctional systems have demonstrated the potential of inorganic NP–hydrogel composites in cranial defect models. For example, GelMA-based hydrogels incorporating mesoporous whitlockite NPs loaded with taxifolin enabled the simultaneous inhibition of osteoclastogenesis while promoting angiogenic and osteogenic responses in osteoporotic cranial defects in ovariectomized (OVX) rats [223]. In another approach, alginate-based hydrogels containing whitlockite particles created a favourable osteogenic niche that supported stem cell-mediated bone regeneration. This system also modulated osteoclast activity through Mg^2+^ ion release, thereby enhancing bone repair in murine calvarial defect models [31].

Sequential delivery strategies have also been developed to better coordinate angiogenic and osteogenic signalling. In one representative system, BMP-2-loaded mesoporous silica NPs were incorporated into a photo-crosslinked composite hydrogel based on aldehyde hyaluronic acid and GelMA, together with VEGF. BMP-2 was released in a sustained manner from the silica NPs, while VEGF was immobilized within the hydrogel network via Schiff–base interactions. This design enabled temporally controlled, sequential pro-angiogenic and osteoinductive signalling, resulting in substantial calvarial defect repair in a rat model [21].

Beyond conventional inorganic NPs, metal–organic frameworks (MOFs) such as ZIF-8 and ZIF-67 have emerged as versatile platforms for drug and ion delivery due to their high porosity and tuneable metal composition. For instance, GelMA hydrogels incorporating ZIF-8 loaded with metformin and Zn^2+^ ions exhibited pronounced anti-inflammatory and osteogenic effects in diabetic cranial defect models, significantly increasing bone volume and matrix deposition. Similarly, PEGDA/ZIF-8 hydrogels loaded with simvastatin demonstrated synergistic lipid-regulatory and osteoinductive effects in hyperlipidemic models, leading to enhanced osteointegration and angiogenesis [221,224].

Magnetic NPs have also been explored to further enhance the functionality of hydrogel systems. In this context, iron oxide (Fe_3_O_4_) NPs embedded within tannic acid-modified silk fibroin hydrogels improved osteogenic differentiation and bone regeneration. The resulting hybrid hydrogel exhibited strong tissue adhesion and excellent biocompatibility, supporting robust bone formation in both in vitro and in vivo models [26]. A recent strategy exploited iron oxide NPs not only as bioactive components but also as multifunctional crosslinkers of GelMA hydrogels. By reinforcing the polymer network while simultaneously providing pro-angiogenic and osteogenic stimuli, these NP-crosslinked hydrogels significantly enhanced vascularisation and cranial bone regeneration in rat calvarial defects, demonstrating how inorganic NPs can simultaneously improve mechanical performance and biological activity [225].

In addition to promoting osteogenesis, inorganic NPs can address specific microenvironmental challenges associated with cranial defects. For example, a thermosensitive Pluronic F127 hydrogel containing oxygen-releasing microspheres (CaO_2_/PCL) and nano-hydroxyapatite/laponite NPs was developed to sustain oxygenation for up to three weeks post-implantation. This system maintained MSCs and endothelial cell viability under hypoxic conditions and enhanced both angiogenesis and bone quality in rat cranial defects, offering a minimally invasive strategy for regeneration under metabolic stress [226].

Infection control represents another critical aspect of cranial implant performance. In this regard, mesoporous silica NPs embedded in PEG-based hydrogels have been used for localised antibiotic delivery. Clindamycin-loaded MSNs enabled prolonged and controlled antimicrobial release, effectively preventing infection-related complications and highlighting the multifunctional potential of inorganic NP–hydrogel systems [227].

More recently, a freeze-dried poly(acrylic acid)/tricalcium phosphate nanoparticle scaffold was used to deliver human umbilical cord MSC-derived exosomes [228]. The porous scaffold released approximately 68% of the loaded exosomes over 14 days and was evaluated using 150 μg of exosomes in 8 mm critical-sized calvarial defects in Wistar rats. At 8 and 12 weeks, micro-CT, histology, and OCN/CD31 immunohistochemistry showed significantly greater osteogenesis and vascularisation in the exosome-enriched group than with the scaffold alone. Specifically, BV/TV, bone mineral density, and trabecular thickness increased by 2.6-, 3.7-, and 2.0-fold, respectively, at 8 weeks and by 2.4-, 2.2-, and 1.7-fold at 12 weeks. However, the mechanisms governing exosome–scaffold interactions were not fully elucidated, and the construct was a freeze-dried scaffold rather than a conventional injectable hydrogel.

Finally, immunomodulatory strategies have also been integrated into inorganic NP–hydrogel systems. Sun and colleagues reported a tetra-PEG composite hydrogel incorporating short-chain chitosan and nano-hydroxyapatite particles for sustained parathyroid hormone (PTH) release. This platform promoted macrophage polarization toward an M2 phenotype, inhibited osteoclast activity, and significantly improved bone regeneration in osteoporotic calvarial defects [229]. Multifunctional inorganic nanomaterials have also been combined with injectable hydrogels to simultaneously regulate infection, oxidative stress, and osteoimmunity. Zhu et al. developed a photoactivated silk fibroin/chitosan hydrogel incorporating MXene nanosheets decorated with ZIF-8 NPs, enabling photothermal antibacterial activity together with stimuli-responsive tannic acid and Zn^2+^ release. This multifunctional platform promoted M2 macrophage polarization, inhibited osteoclastogenesis, enhanced angiogenesis and osteogenesis, and significantly accelerated diabetic calvarial bone regeneration [272].

##### Organic NPs

Organic NPs, including chitosan-based systems, poly(lactic-co-glycolic acid) (PLGA), and lipid-derived nanocarriers, are widely integrated into hydrogel matrices as biodegradable secondary delivery platforms for cranial bone regeneration. Their chemical tunability and compatibility with hydrophobic or labile bioactive agents enable the design of sequential, dual, or stimuli-responsive release systems. These features enhance therapeutic precision while preserving hydrogel injectability and structural integrity. As a result, organic NP–hydrogel composites have emerged as versatile platforms capable of modulating inflammation, stem cell recruitment, gene expression, and osteogenic differentiation in cranial defect models.

Several multifunctional systems have demonstrated coordinated immunomodulatory and osteogenic effects. For instance, an injectable photocrosslinkable GelMA/heparin-modified hyaluronic acid hydrogel containing IL-10 and icariin-loaded PLGA–HA nanoparticles enabled sequential immunomodulatory and osteogenic stimulation. IL-10 was released rapidly, reaching 21.9% after 1 day and 63.1% after 7 days, whereas icariin showed sustained release up to 83.7% at day 28. In vitro, the dual-delivery system promoted M2 polarization of RAW264.7 macrophages and enhanced the osteogenic differentiation and matrix mineralization of rat BMSCs. In bilateral 5 mm critical-sized rat calvarial defects, micro-CT and histological analyses at 8 weeks showed the highest BV/TV and bone mineral density, together with almost complete defect filling, in the combined IL-10/icariin group. However, validation was limited to a short-term small-animal model, and the complexity of the multicomponent photocrosslinked system may hinder clinical translation [84]. This spatiotemporally programmed strategy significantly enhanced bone remodelling in rat critical-sized calvarial defects, underscoring the importance of sequential immune and osteogenic regulation for high-quality regeneration.

In a complementary approach, an injectable thermosensitive chitosan/β-glycerophosphate hydrogel was combined with chitosan/carboxymethyl chitosan nanoparticles carrying SDF-1α [231]. The nanoparticles exhibited a loading efficiency of 85.1 ± 7.7% and reduced the initial burst release of the chemokine: whereas free SDF-1α in the hydrogel reached approximately 85% release within 4 days, the nanoparticle-containing system released only 40% over 28 days. In vivo, the formulation was evaluated in 8 mm critical-sized calvarial defects in Sprague–Dawley rats and assessed after 8 weeks by micro-CT and histology. The nanoparticle–hydrogel group achieved 38.5 ± 4.5% new bone formation, compared with 26.3 ± 7.25% for hydrogel containing free SDF-1α and 8.64 ± 4.8% for untreated defects. These findings support the use of nanoparticle-containing injectable hydrogels for prolonged chemokine delivery and endogenous cell recruitment, although the study did not directly quantify MSC homing and was limited to a short-term small-animal model [231].

Lipid-based nanocarriers have also been successfully employed for the delivery of hydrophobic bioactive molecules. In a study, solid lipid NPs (SLNs) encapsulating RESV were incorporated into GelMA hydrogels to form a Res-SLNs/GelMA scaffold. This system provided sustained and controlled release of Res, significantly enhancing osteogenic differentiation of BMSCs and promoting bone regeneration in rat cranial defect models. The scaffold exhibited favourable osteoconductive and osteoinductive properties, demonstrating the effectiveness of lipid-based NPs for hydrophobic drug delivery within hydrogel systems [232].

Stimuli-responsive organic NPs have further expanded the functionality of these composite systems. Thermosensitive chitosan/poly(N-isopropyl acrylamide) NPs were developed for the delivery of simvastatin acid and incorporated into electroactive silk fibroin–polyacrylamide hydrogels containing vitamin C. The resulting composite exhibited appropriate gelation behaviour, mechanical stability, and temperature-dependent drug release, with accelerated release at physiological conditions. In aged rat calvarial defect models, this dual-loaded hydrogel significantly enhanced both bone regeneration and vascularisation, highlighting its potential for treating age-related bone defects [32].

Song et al. developed an alginate-based hydrogel embedding nicaraven-loaded chitosan NPs. The hybrid system displayed favourable swelling and degradation profiles, along with high cytocompatibility and controlled release behaviour in vitro. In vivo, the composite significantly improved cranial bone repair and upregulated the expression of collagen type II and TGF-β3, supporting its application in osteochondral regeneration [233].

Gene delivery strategies have also been explored using organic NP–hydrogel systems. A thermosensitive hydrogel incorporating chitosan NPs loaded with BMP-2 plasmid DNA (pDNA-BMP-2) enabled sustained release of genetic material, promoting osteoinduction and enhancing endogenous bone regeneration. The combination of thermoresponsive gelation and gene-loaded NPs facilitated localised and prolonged delivery, representing a promising approach for cranial bone defect repair [160].

##### Organic/Inorganic Hybrid NPs

Organic–inorganic hybrid NPs combine the biocompatibility and tuneable degradation of organic polymers with the mechanical strength, stability, and functional versatility of inorganic components. When incorporated into hydrogel matrices, hybrid NPs support the development of multifunctional platforms capable of localised therapeutic delivery while simultaneously providing imaging or microenvironment-responsive features.

In an innovative study, a multifunctional cell-laden hydrogel strategy combined intracellular delivery of resveratrol-loaded PLGA/iron oxide NPs with non-invasive tracking of transplanted MSCs. After pre-loading the cells, MSCs were encapsulated within PEG-based hydrogels, resulting in enhanced osteogenic matrix deposition and improved cranial bone repair in mouse models. Importantly, the iron oxide component enabled real-time photoacoustic monitoring of the implanted cell population, illustrating how hydrogel-based cell delivery platforms can integrate therapeutic and imaging functions within a single construct [234].

More recently, a pH-responsive chitosan-coated mesoporous silica NP system loaded with naringin was incorporated into GelMA hydrogels to achieve microenvironment-sensitive drug release. The chitosan shell acted as a gatekeeper, allowing for preferential release under acidic conditions associated with osteoclastic activity. This hybrid platform effectively suppressed osteoclast formation while promoting new bone formation in rat calvarial defects, demonstrating the capacity of organic–inorganic composites to simultaneously modulate anabolic and anti-resorptive pathways during cranial bone repair [235].

These hybrid nanostructures represent a new generation of secondary delivery systems that bridge material engineering with regenerative medicine, opening new perspectives for theranostic bone repair in complex craniofacial settings.

#### 3.3.3. EVs-Integrated Hydrogel

EVs, particularly exosomes, have emerged as promising cell-free therapeutic agents in regenerative medicine due to their biocompatibility, stability, and intrinsic paracrine signalling capacity. Produced by a wide range of cell types through the endosomal pathway, EVs carry bioactive cargos including proteins, lipids, mRNAs, and microRNAs that can recapitulate many therapeutic functions of their parent cells while avoiding the immunological and safety concerns associated with direct cell transplantation [273]. Their nanoscale dimensions and lipid membrane composition facilitate cellular uptake and local signalling, making them particularly suitable for localised delivery in cranial bone regeneration. Incorporation within hydrogel matrices further improves EV stability and retention at the defect site, enabling sustained and spatially controlled release that modulates osteogenesis, angiogenesis, and immune responses during bone repair.

Several studies support the efficacy of EV-loaded hydrogels in cranial models. Hydrogels delivering BMP-2-enriched exosomes significantly enhanced MSC osteogenic differentiation and accelerated bone formation in a rat calvarial defect model [236]. Similarly, GelMA hydrogels delivering exosomes derived from BMP-2-overexpressing NIH-3T3 fibroblasts preserved the osteoinductive activity of the engineered vesicles and enhanced BMSC differentiation, resulting in robust mice cranial bone regeneration in vivo [237]. A composite GelMA–hyaluronic acid methacrylate/nano-hydroxyapatite (GelMA-HAMA/nHAP) hydrogel loaded with urine-derived stem cell exosomes (USC-EXOs) further demonstrated coordinated stimulation of BMSC osteogenesis and endothelial cell-mediated angiogenesis; implantation in rat cranial defects resulted in extensive bone regeneration accompanied by formation of H-type vessels [238].

Engineered EV delivery strategies have further expanded the functionality of these systems. Curcumin-loaded MSC-derived exosomes encapsulated within bisphosphonate-modified GelMA microspheres provided sustained release of both antioxidant molecules and EV-derived signals. This system enhanced M2 macrophage polarization, suppressed osteoclast activity, and promoted BMSC proliferation and osteogenic differentiation, ultimately accelerating the closure of rat critical-sized calvarial defects [239]. A sequential delivery strategy was developed using an injectable thermosensitive hydrogel composed of 20% PLGA–PEG–PLGA, freely incorporated VEGF (200 ng mL^−1^), and chitosan nanofibrous microspheres loaded with DPSC-derived exosomes (5 mg mL^−1^ CM/Exo) [240]. The hydrogel underwent sol–gel transition at approximately 28 °C, enabling injection and rapid in situ gelation at physiological temperature. Differential cargo compartmentalization produced an early angiogenic phase followed by sustained osteogenic stimulation: approximately 70% of VEGF was released within the first 7 days and 96% by day 30, whereas only 9.9% of the exosomes was released during the first week, followed by cumulative release of approximately 87.4% over the subsequent three weeks. In vitro, VEGF-containing formulations enhanced HUVEC tube formation and increased PDGF-β and Ang-1 expression, while DPSC-Exo-loaded microspheres promoted MC3T3-E1 osteogenic differentiation, as demonstrated by ALP activity, matrix mineralization, and increased RUNX2, BMP-2, and osteocalcin expression. In vivo efficacy was assessed in bilateral 5 mm critical-sized calvarial defects in male Sprague–Dawley rats using five experimental groups (*n* = 5), with 100 μL of hydrogel injected into each defect. After 8 weeks, micro-CT analysis showed that the dual-delivery group exhibited the highest bone volume and BV/TV and the smallest residual defect diameter, while H&E, Masson’s trichrome, and CD31 immunostaining confirmed extensive new bone formation and enhanced vascularisation. Although this programmed-release strategy more closely reproduced the physiological sequence of angiogenesis followed by osteogenesis than single-cargo formulations, residual chitosan microspheres were still observed at 8 weeks, and validation was limited to a short-term small-animal model. Gene-engineered vesicles have been investigated as well; exosomes enriched with osteoinductive *miR-26a* enhanced cranial bone regeneration by simultaneously activating angiogenic pathways and upregulating osteogenic gene expression while suppressing osteoclast-associated signalling in OVX rats [236,241]. Exosome-mediated delivery of osteogenic miRNAs has also been explored, with sustained release systems enhancing in vivo cranial bone formation [242].

Strategies aimed at improving EV scalability and bioactivity have also been developed. Extrusion-derived exosome mimetics produced from genetically modified MSCs exhibited enhanced osteogenic properties and, when delivered within injectable chitosan hydrogels, promoted substantial regeneration of nonhealing calvarial defects in mouse models [243]. Hypoxia preconditioning of MSCs represents another approach to enhance EV functionality. Hypoxia-derived EVs incorporated into PEG/polypeptide hydrogels displayed sustained release and significantly enhanced osteoblast proliferation, migration, and mineralization through activation of PI3K/Akt signalling in a rat cranial model [273]. Similarly, exosome mimetics derived from hypoxia-conditioned BMSCs demonstrated superior pro-angiogenic and osteogenic activity compared with native EVs when applied to rat cranial critical-sized model [274].

Combinatorial delivery strategies integrating EVs with inorganic components have also shown synergistic regenerative effects. MSC-derived exosomes incorporated into ZIF-8-modified GelMA hydrogels enabled coordinated osteogenesis, angiogenesis, and immunomodulation through the sustained release of EV cargos together with Zn^2+^ ions. In rat cranial defect models, this composite system significantly enhanced bone regeneration while reducing local inflammation by promoting macrophage M2 polarization and activating osteogenic signalling pathways in BMSCs [275]. More recently, hybrid EV systems have been developed to further enhance EV stability and osteoinductive activity. Wu et al. engineered calcium phosphate NP-coated M2 macrophage-derived extracellular vesicles (M2EV@CaP) and incorporated them into a hydrogel for localised delivery. The inorganic shell protected EV integrity while providing inflammation-responsive calcium/phosphate ion release, promoting M2 macrophage polarization, stem cell osteogenic differentiation through Ca^2+^/Akt signalling, and significantly enhancing diabetic calvarial bone regeneration in vivo [276]. Engineered EVs have recently been functionalized to further enhance their therapeutic efficacy. Yao et al. developed mitochondria-targeted BMSC-derived exosomes carrying the antioxidant MitoQ and functionalized with a BMSC-targeting aptamer before incorporation into a SIS-coated scaffold. This multifunctional strategy reduced mitochondrial oxidative stress while simultaneously enhancing stem cell recruitment, angiogenesis, osteogenesis, and calvarial bone regeneration [277].

Among acellular therapeutic strategies, EV-loaded hydrogels represent one of the most promising emerging approaches because they retain many of the regenerative and immunomodulatory functions of stem cells while avoiding several safety concerns associated with cell transplantation. However, standardized protocols for EV isolation, characterisation, dosage, and large-scale production remain lacking, representing one of the principal barriers to clinical translation.

#### 3.3.4. MPs-Integrated Hydrogels

MPs, including microspheres as one of the most widely used subclasses, have emerged as versatile secondary delivery systems for hydrogel-based drug delivery to achieve controlled, prolonged, or sequential release of bioactive agents in cranial bone regeneration. By dispersing polymeric or inorganic microspheres within hydrogel matrices, these composites mitigate burst release and create localised reservoirs for hydrophobic drugs, proteins, growth factors, and nucleic acids, thereby enhancing therapeutic precision and temporal regulation [66].

In particular, microsphere-based systems have been widely explored to address key microenvironmental limitations of cranial defects, such as hypoxia and insufficient vascularisation [244]. Multiple strategies have demonstrated their regenerative potential in cranial models. Porous carbonate hydroxyapatite microspheres co-loaded with RESV and DEX promoted osteogenesis and immunomodulation in osteoporotic defects, while oxygen- and magnesium-releasing microspheres improved angiogenesis and mineralization under hypoxic conditions [245]. Similarly, calcium peroxide-loaded polycaprolactone microspheres supported sustained oxygen delivery, enhancing cell survival and bone formation in rat calvarial defect model [246].

Beyond microenvironmental regulation, microsphere incorporation enables precise control over growth factor delivery and release kinetics. To achieve more precise growth factor control, an injectable alginate hydrogel containing BMP-2-loaded recombinant collagen microspheres enabled spatiotemporal delivery of BMP-2, reducing the total dose required to induce effective osteogenesis. In rat calvarial models, complete defect bridging was achieved within eight weeks, demonstrating sustained BMP-2 release and enhanced bone formation [247].

Likewise, Lou et al. developed a spatiotemporally graded hydrogel system comprising a GelMA matrix encapsulating mesenchymal and endothelial cells together with dimethyloxalylglycine (DMOG), combined with silk fibroin microspheres loaded with nano-hydroxyapatite [248]. Differential degradation kinetics enabled early DMOG release to promote prevascular network formation, followed by sustained nHAp presentation to support osteogenic maturation. During 21 days of dynamic in vitro culture, the system synchronized vascular perfusion with bone matrix deposition. In mouse critical-sized calvarial defects, the sequential angiogenic–osteogenic strategy produced significantly greater bone regeneration and functional microvascular integration than static scaffold controls. Nevertheless, the incorporation of multiple biomaterials, therapeutic cues, and cell populations increases manufacturing complexity and may hinder clinical translation.

In addition to controlled release, recent studies have increasingly focused on the development of multifunctional microsphere-based systems capable of simultaneously modulating multiple regenerative pathways. Recent microfabrication approaches have improved structural precision and multifunctionality. Polydopamine-functionalized porous PLGA microspheres incorporated into GelMA hydrogels containing tobramycin and hydroxyapatite NPs exhibited antibacterial, antioxidative, and osteoinductive effects in rat cranial defects [249]. Similarly, silk fibroin-based methacrylated hydrogels embedding platelet-rich plasma and berberine-loaded microspheres enhanced stem cell recruitment and osteogenesis even under inflammatory conditions in rat calvarial defects [223].

Furthermore, advanced designs are now incorporating external or stimuli-responsive control to better mimic the dynamic nature of bone healing. Photothermal-responsive poly(N-acryloyl glycinamide-co-acrylamide) microspheres containing indocyanine green and parathyroid hormone embedded in a poly(dimethylaminoethyl methacrylate-co-2-hydroxyethyl methacrylate) hydrogel enabled both pulsatile and sustained hormone release, stimulating osteoblast and osteoclast activity and restoring bone mass in OVX rats [250].

Another critical aspect of these systems is their ability to support long-term and sustained therapeutic delivery. Moreover, PLGA/nanohydroxyapatite microspheres incorporated into dopamine-modified hyaluronic acid/collagen I hydrogels formed highly interconnected porous scaffolds that enhanced stem cell infiltration, angiogenesis, and cranial bone regeneration in mouse models [251].

Long-term controlled release platforms have also been explored. DEX-loaded mesoporous silica/PLGA microspheres embedded within a methacrylated silk fibroin–alginate hydrogel sustained drug delivery for over four months, improving MSC osteogenesis, macrophage M2 polarization, and angiogenesis in rat calvarial defects [245]. Similarly, oxygen-releasing gelatin–calcium peroxide MPs integrated into self-assembling peptide hydrogels and PLGA membranes extended oxygen availability for up to four weeks, enhancing transplanted cell viability and promoting bone formation in critical-sized rat calvarial defect models [49].

Compared with NPs, MPs generally allow for higher drug loading and prolonged release over extended periods, making them particularly suitable for sustained delivery of growth factors and other macromolecules. However, their larger size may reduce injectability and limit homogeneous distribution within hydrogel matrices, requiring careful optimization according to the intended regenerative application.

#### 3.3.5. Two-Dimensional (2D) Nanomaterial-Integrated Hydrogel

The mechanical and biochemical properties of hydrogel-based delivery systems play a crucial role in guiding stem cell behaviour during cranial bone regeneration. Specifically, the stiffness, viscoelasticity, and degradation rate of the hydrogel matrix can directly influence the migration, proliferation, and osteogenic differentiation of MSCs. Therefore, engineering hydrogels with optimized mechanical performance and bioactivity is essential to mimic the ECM and accelerate the healing of cranial defects.

In this context, the incorporation of nanostructured materials has emerged as an effective strategy to further enhance hydrogel functionality. 2D nanomaterials, such as black phosphorus (BP), MXene, and graphene oxide (GO), have emerged as versatile additives to improve both the structural and functional performance of hydrogels [54,278]. These nanosheets offer a high surface-to-volume ratio, tuneable electronic and chemical properties, and intrinsic biological activities, making them suitable platforms for drug delivery, cell modulation, and tissue integration.

Among these materials, black phosphorus has attracted particular attention due to its multifunctional bioactivity. BP nanosheets exhibit photothermal, immunomodulatory, and neurogenic properties that can promote both angiogenesis and osteogenesis [220]. Their incorporation into hydrogel matrices also enables externally triggered therapeutic functions, including antibacterial activity and stimulus-responsive drug release, while supporting nerve regeneration in cranial repair models.

Beyond BP-based systems, graphene-derived nanomaterials have been investigated for their ability to improve both the mechanical and biological performance of composite scaffolds. Tavakoli et al. fabricated a 3D-printed polylactic acid/hardystonite–graphene oxide (PLA/HTGO) framework whose channels were filled with a freeze-dried pectin-quaternized chitosan polyelectrolyte phase containing 20 mg mL^−1^ simvastatin (FD-Sim) [279]. A dip-coated simvastatin-loaded formulation (DC-Sim) was also prepared for comparison. The resulting constructs exhibited interconnected macro- and microporosity, while simvastatin release after 28 days reached 86.02 ± 3.63% from FD-Sim and 77.40 ± 5.25% from DC-Sim. In vitro, MG-63 cells showed greater proliferation, attachment, and spreading on the simvastatin-containing scaffolds, with the strongest response observed for FD-Sim. In a rat critical-sized calvarial defect model, the FD-Sim scaffold promoted almost complete defect repair after eight weeks, with the defect largely filled by newly formed bone. These findings highlight the advantage of combining a mechanically reinforced 3D-printed framework with a highly porous drug-loaded polymeric phase; however, the construct is more appropriately regarded as a composite scaffold than as a conventional injectable hydrogel, which should be considered when comparing it with hydrogel-based delivery systems.

Similarly, MXene-based nanocomposite hydrogels have emerged as promising candidates due to their unique physicochemical properties and bioactivity. An injectable gelatin methacrylate/alginate–dopamine hydrogel incorporating copper-decorated Ti_3_C_2_Tx MXene nanosheets formed an interpenetrating network through covalent and noncovalent interactions, providing improved mechanical strength, self-healing behaviour, and tissue adhesion. The gradual release of Cu^2+^ ions stimulated osteogenic differentiation and angiogenic activity, and implantation in diabetic rat cranial defects resulted in enhanced bone regeneration and vascularisation, together with modulation of the local inflammatory microenvironment [280].

Additional strategies have further expanded the versatility of 2D nanomaterials by combining them with bioactive molecules and advanced fabrication approaches. For example, a Tetra-PEG/silk fibroin hydrogel incorporating GO nanosheets loaded with icariin formed a semi-interpenetrating polymer network enabling sustained drug release for approximately two weeks. The system promoted rBMSC osteogenic differentiation in vitro and significantly accelerated bone regeneration in a mouse calvarial defect model, as demonstrated by micro-CT and histological evaluation [85]. Similarly, the incorporation of GO nanosheets into 3D-bioprinted alginate/gelatin scaffolds increased compressive modulus and improved mineralization in hMSC-laden constructs, indicating enhanced structural stability and osteogenic potential [36].

In addition to graphene-based systems, nanoclay-based hydrogels have demonstrated unique advantages, particularly in compromised or metabolically impaired environments. GelMA hydrogels incorporating LAPONITE^®^ nanosheets and platelet-derived growth factor-BB (PDGF-BB) provided mechanical reinforcement together with sustained growth factor delivery. The nanoclay released bioactive ions such as Si^4+^, Mg^2+^, and Li^+^, which helped restore stem cell function and supported angiogenesis under hyperglycemic conditions. In diabetic rat calvarial defects, this nanocomposite hydrogel promoted robust bone regeneration and improved vascularisation compared with growth factor–free controls [281].

#### 3.3.6. Stimuli-Responsive Hydrogel Platforms

Stimuli-responsive hydrogels represent a dynamic extension of secondary delivery systems, enabling on-demand therapeutic release in response to endogenous or externally applied triggers. These platforms bridge static scaffold design and dynamic tissue regeneration, representing a promising direction for precision-guided craniofacial repair. By sensing variations in pH, redox state, enzymatic activity, or inflammatory signals, or by responding to external stimuli such as near-infrared (NIR) irradiation, ultrasound, magnetic fields, or temperature, these systems achieve spatially confined and temporally synchronized drug delivery [282,283,284,285,286]. This adaptive behaviour is particularly advantageous in cranial bone repair, where fluctuating inflammatory and metabolic conditions require tightly regulated therapeutic modulation [271].

Among external stimuli, NIR-responsive systems have been extensively investigated due to their non-invasive controllability and precise spatiotemporal activation. For instance, NIR-triggered BP/Mg^2+^-GelMA hydrogels achieved photothermal antibacterial effects and enhanced neurogenic bone healing in rat infected cranial defect models, while chitosan hydrogels containing polydopamine-functionalized magnesium carbonate microspheres exhibited sequential aspirin and BMP-2 release upon NIR irradiation, promoting vascularised bone regeneration [220]. Similarly, MXene/PVA hydrogels enabled NIR-controlled DEX release, effectively modulating local inflammation and facilitating bone matrix formation in cranial reconstructions. Another example is the reduced graphene oxide (rGO)-loaded chitosan hydrogel designed for NIR-triggered release of teriparatide in osteoporotic cranial defects. The photothermal conversion capability of rGO enabled pulsatile, physiologically relevant delivery patterns of the anabolic peptide, stimulating osteogenesis and angiogenesis in vivo and demonstrating the clinical potential of NIR-responsive systems for osteoporotic bone repair [287]. NIR-responsive hydrogels have also been exploited to dynamically regulate the paracrine activity of encapsulated cells rather than simply controlling cargo release. A recent ECM-mimetic dynamic fibrous hydrogel combined photothermal responsiveness with antioxidant protection to enhance MSC survival and stimulate SDF-1 secretion under NIR irradiation, thereby promoting endogenous stem cell recruitment, angiogenesis, and cranial bone regeneration in murine cranial defects [288].

In addition to NIR-triggered systems, other externally activated platforms have been developed to further refine temporal control over therapeutic delivery. For instance, poly(dimethylaminoethyl methacrylate-co-2-hydroxyethyl methacrylate) hydrogels embedded with parathyroid hormone-loaded microspheres exhibited NIR-triggered gel–sol transitions, enabling on-demand hormone delivery while generating micropores that supported osteoblast and osteoclast activity, thus restoring bone remodelling balance in OVX rats [250].

Alternative strategies have exploited ultrasound, biochemical cues, and magnetic stimulation to achieve similar levels of control. Ultrasound-sensitive alginate or GelMA-heparin matrices have been used to spatially and temporally regulate the release of growth factors and oxygen, thereby accelerating angiogenesis and mineralization [286]. Furthermore, redox- and enzyme-responsive hydrogels have been developed to deliver antimicrobial and osteogenic agents specifically within chronic inflammatory cranial defects, reducing pro-inflammatory cytokine expression and promoting tissue repair [270,289]. Magnetically responsive hydrogels composed of regenerated silk fibroin, tannic acid, and Fe_3_O_4_ NPs further expand this concept by allowing for shape adaptability and rapid gelation under magnetic fields. The magnetic stimulation activated the cGMP/PKG/ERK signalling pathway, promoting osteogenic differentiation and accelerating bone regeneration in rat critical-sized cranial defects [26].

Temperature-responsive systems represent another important class of stimuli-responsive hydrogels, particularly for minimally invasive delivery. Xu et al. developed an injectable thermosensitive hydrogel composed of 20% w/v Pluronic F127, CaO_2_-loaded PCL microspheres, nano-hydroxyapatite, and laponite [246]. The temperature-induced sol–gel transition enabled minimally invasive injection and in situ retention, whereas the PCL microspheres prolonged oxygen generation for up to 21 days, thereby mitigating local hypoxia and supporting osteogenic and angiogenic responses. In vitro, the system was evaluated using BMSCs and endothelial cells under hypoxic conditions through cell viability, migration, tube-formation, ALP activity, matrix mineralization, and osteogenic-marker analyses. In vivo efficacy was assessed in an 8 mm critical-sized rat calvarial defect at 4 and 8 weeks using micro-CT, histological analysis, and osteogenic and vascular immunostaining. Compared with formulations lacking either the oxygen-releasing microspheres or the osteoinductive nanoparticles, the complete system significantly increased bone volume fraction, trabecular thickness and number, and vascular density, while reducing trabecular separation (*p* < 0.05). Despite these promising findings, validation was limited to an eight-week small-animal model, and long-term material clearance and oxygen-related safety remain to be established.

A similar thermosensitive chitosan-based hydrogel (CS/CSn-GP) enables in situ gelation and supports cell adhesion and matrix deposition, with in vivo studies confirming accelerated bone formation and defect closure in calvarial defects [160]. Likewise, thermoresponsive poloxamer hydrogels have served as injectable scaffolds capable of structural reinforcement and localised delivery of bioactive agents, particularly when combined with drug-loaded microspheres, leading to significantly enhanced bone regeneration [290].

## 4. Conclusions and Future Directions

The persistent clinical difficulty in achieving stable and biologically integrated reconstruction of critical-sized cranial defects underscores the limitations of conventional grafting and synthetic implants. Although structural closure can be obtained, predictable regeneration of vascularised and remodelled bone within the confined cranial niche remains challenging. Unlike long bones, the calvarium is not exposed to significant mechanical loading, yet it requires strict dimensional stability, precise defect conformity, and preservation of intracranial structures. At the same time, critical-sized cranial defects are frequently characterised by limited intrinsic vascularity, spatial restriction, persistent inflammation, and heterogeneous oxygen distribution. These conditions create a biologically compromised microenvironment in which spontaneous healing is minimal and regenerative events must be tightly coordinated in space and time.

Within this setting, localised therapeutic delivery emerges not simply as a technical refinement but as a fundamental requirement. Systemic administration of osteoinductive molecules is constrained by rapid clearance and off-target effects, while direct bolus application fails to provide sustained bioactivity during the sequential phases of healing. The hydrogel-based strategies analysed in this review address this gap by combining structural adaptability with the programmable release of bioactive signals. Hydrogel injectability and conformability enable intimate adaptation to irregular cranial defects, whereas tuneable crosslinking density, degradation kinetics, and diffusion properties allow for alignment between scaffold resorption and tissue formation. To date, the vast majority of hydrogel-based drug delivery strategies for cranial bone regeneration remain at the preclinical stage, with only limited clinical evidence available, highlighting the need for well-designed translational studies.

Across preclinical in vivo cranial defect models, different hydrogel-mediated delivery strategies have consistently demonstrated enhanced angiogenesis, osteogenic differentiation, and defect bridging. Rather than acting through isolated pathways, these systems converge on a common principle: effective cranial regeneration requires coordinated immuno-vascular-osteogenic signalling. Hydrogels provide a versatile framework for implementing staged, synergistic, or combinatorial delivery strategies. Natural hydrogels exhibit excellent biocompatibility but limited mechanical properties, whereas synthetic hydrogels provide greater tunability at the expense of bioactivity. Hybrid systems increasingly combine these complementary features and currently represent the most promising approach. However, current evidence does not support the existence of a universally superior hydrogel platform. Instead, therapeutic performance appears to depend on the specific biological objective (e.g., immunomodulation, angiogenesis, osteogenesis, or combined regeneration), the delivered cargo, and the pathological context of the cranial defect. Similarly, growth factors remain the most extensively investigated therapeutic agents, whereas nucleic acids and extracellular vesicles offer higher biological specificity but require more sophisticated delivery systems. Overall, multifunctional hydrogels integrating immunomodulatory, angiogenic and osteogenic cues appear to achieve the most robust regenerative outcomes in preclinical cranial defect models. A direct comparison among these platforms remains challenging due to the heterogeneity of hydrogel formulations, animal models, defect dimensions, and outcome measures. Moreover, differences in defect size, species, follow-up duration, methods used to quantify bone regeneration (micro-CT parameters, histomorphometry, immunohistochemistry), and release protocols often lead to apparently inconsistent outcomes, making it difficult to identify whether superior regenerative performance arises from the hydrogel composition itself, the delivered therapeutic agent, or the experimental design. Nevertheless, from a near-term translational perspective, injectable, biodegradable, and acellular hydrogels delivering well-characterised bioactive ions, peptides, or small molecules appear particularly attractive. These systems combine defect conformity and controlled release with relatively simple manufacturing processes, thus circumventing the viability, donor variability, storage, and regulatory issues associated with transplanted cells [54,90,97,98,101,177]. EV-loaded hydrogels have the potential to offer a more extensive biological effect by simultaneously regulating immune responses, angiogenesis, and osteogenesis while maintaining a cell-free format. However, the advancement of these hydrogels into clinical practice remains contingent on the standardisation of EV isolation, characterisation, potency assessment, and scalable production [236,237,239,240,241,273,278]. The integration of NPs into hydrogels, along with their responsiveness to specific stimuli, has emerged as a promising approach for precision delivery. This approach holds particular promise for applications in infected, inflammatory, or metabolically compromised defects. However, it is important to note that the increased complexity in composition and manufacturing processes may impede reproducibility and regulatory acceptance [84,232,252,256,271,282,284]. Cell-laden hydrogels remain among the most biologically potent approaches, particularly for large or compromised defects, but currently carry the greatest translational burden [187,284]. Consequently, the most promising systems may not be the most compositionally complex, but rather those that achieve coordinated immunomodulatory, angiogenic, and osteogenic effects through reproducible release and degradation profiles using the minimum necessary number of components.

Recent studies further illustrate emerging directions in hydrogel-based cranial bone regeneration. Chronologically programmed biomimetic niches have been developed to provide an early phase of immunomodulation, endogenous progenitor recruitment, and vascular activation, followed by a later osteogenic phase triggered by progressive hydrogel degradation. This type of stage-specific delivery is particularly relevant in metabolically compromised environments, such as diabetic cranial defects, where restoration of endogenous regenerative activity is essential. In parallel, extrusion-printed alginate–gelatin hydrogels containing amorphous magnesium phosphate show how bioactive ion delivery can be combined with printability, controlled degradation, and defect-specific scaffold fabrication. Although these 3D-printed systems still require in vivo validation, they highlight the potential of integrating biological programming with reproducible and patient-specific manufacturing [291,292].

Despite the wealth of promising experimental evidence, several obstacles continue to limit clinical translation. Clinical safety requires rigorous evaluation of both the hydrogel matrix and the delivered cargo, including immunogenicity, cytotoxicity, uncontrolled release, infection risk, and potential adverse interactions with adjacent cranial tissues [54,256]. Translation to Good Manufacturing Practice-compliant production also requires batch-to-batch reproducibility, scalable manufacturing, adequate storage stability, and sterilization procedures that preserve hydrogel structure, mechanical properties, cargo bioactivity, and release kinetics [293,294]. Regulatory pathways become particularly complex for combination products incorporating cells, EVs, NPs, or nucleic acid therapeutics. Finally, long-term studies should establish whether hydrogel degradation and clearance are appropriately synchronized with vascularised bone formation and do not induce chronic inflammation or loss of structural stability [54,293]. In addition, the lack of standardized cranial defect models, heterogeneous follow-up times, and inconsistent outcome measures currently hampers direct comparison across studies and slows identification of clinically relevant design rules.

**Figure 3:**
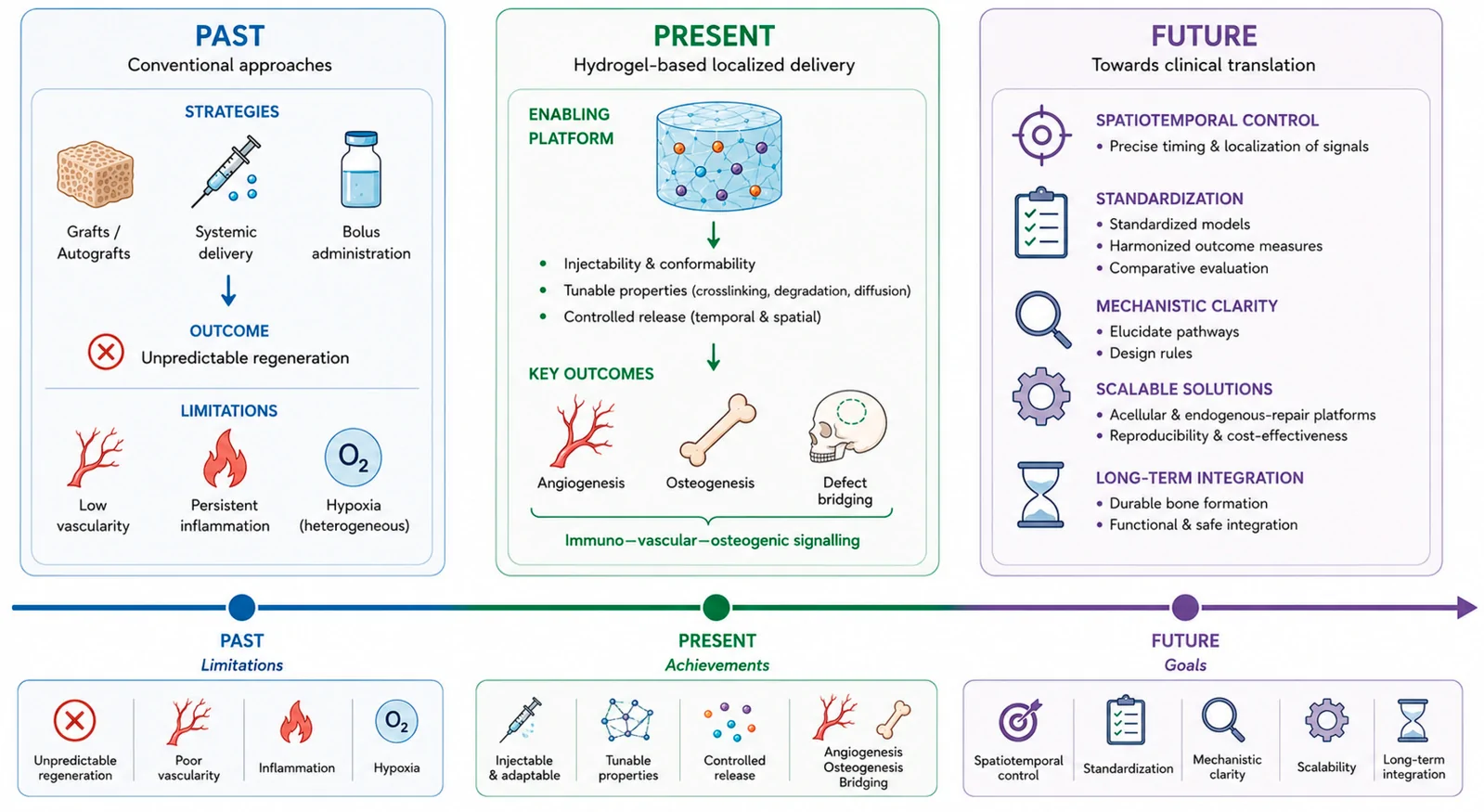
Timeline of current advances and future directions in hydrogel-based cranial bone regeneration. The roadmap summarizes the evolution from conventional grafting, systemic therapy, and bolus administration, which provide limited control over local regenerative processes, to current hydrogel-based platforms offering injectability, defect conformity, localised retention, and controlled therapeutic release. Current outcomes include improved angiogenesis, osteogenesis, immunomodulation, and defect bridging. Future translational priorities include precise spatiotemporal delivery, standardized preclinical models and outcome measures, greater mechanistic clarity, scalable and reproducible manufacturing, and validation of long-term degradation and functional integration. The arrow indicates the temporal progression from conventional approaches to current hydrogel-based platforms and future translational directions.

While cell-laden hydrogels offer strong regenerative potential, their translational burden remains substantial. Conversely, acellular and endogenous-repair-oriented platforms, including EV-loaded, ion-releasing, and stimuli-responsive systems, may offer more scalable routes toward clinical implementation, provided that their release mechanisms and long-term biosafety are rigorously validated. This transition from current hydrogel-based strategies to future translational priorities is schematically summarized in Figure 3.

In conclusion, it is evident that hydrogel-based delivery systems constitute a versatile and adaptable strategy for cranial bone regeneration. Future progress in this field should prioritize the development of injectable, biodegradable, and preferably acellular platforms that can achieve coordinated biological activity with a minimum of compositional complexity. It is imperative to pay particular attention to reproducible release and degradation profiles, which should be synchronized with the inflammatory, angiogenic, osteogenic, and remodelling phases of cranial healing. In order to facilitate meaningful comparison among systems, there is a necessity for the establishment of standardized defect models, the implementation of follow-up periods, and the utilization of quantitative outcome measures, in conjunction with validation in large-animal and clinically compromised models. In order to translate these platforms into clinically applicable therapies, it will be essential to make direct comparisons with current clinical treatments, to consider scalable manufacturing and sterilization requirements at the earliest stage, and to undertake long-term evaluations of vascularised bone quality, material clearance, neural safety, and functional integration.

Overall, no single hydrogel platform currently emerges as universally superior for cranial bone tissue engineering. Rather, the most promising systems are those capable of integrating multiple complementary functionalities, including controlled spatiotemporal drug release, mechanical support, immunomodulation, and promotion of osteogenesis and angiogenesis. Injectable acellular hydrogels incorporating secondary delivery systems currently appear among the most clinically translatable approaches because they combine manufacturing reproducibility with reduced regulatory complexity. Conversely, although cell-laden hydrogels often demonstrate superior regenerative efficacy in preclinical studies, their widespread clinical implementation remains challenged by manufacturing, storage, and regulatory constraints. Future research should therefore move beyond the continuous development of increasingly complex biomaterials and instead prioritize rigorous comparative studies, standardized experimental protocols, clinically relevant animal models, and scalable manufacturing strategies capable of identifying which hydrogel designs truly provide meaningful translational advantages. Ultimately, successful clinical translation will depend not only on maximizing regenerative efficacy, but also on achieving reproducibility, manufacturability, regulatory feasibility, and demonstrable superiority over current standards of care.

## Figures and Tables

**Table 1:** Hydrogel application in BTE. Overview of the main classes of hydrogels used in BTE, including natural, synthetic, semi-synthetic, and hybrid/composite systems. For each category, representative materials, key physicochemical and biological properties, main limitations, and typical applications are summarized.

Hydrogel Type	Representative Materials	Key Advantages	Main Limitations	Typical Applications in Cranial BTE	References
Natural hydrogels	Collagen, gelatin, alginate, chitosan, hyaluronic acid, dextran, fibrin, silk	High biocompatibility, intrinsic bioactivity, structural similarity to the ECM, support for cell adhesion and remodelling	Limited mechanical strength, rapid or variable degradation, batch variability	Cell delivery, growth factor delivery, ECM-mimicking scaffolds for bone regeneration	[38,39]
Synthetic hydrogels	PEG, PVA, PHEMA, PPF, poly(L-glutamic acid)	Highly tuneable physicochemical properties, controllable stiffness and degradation, reproducible network architecture	Lack of intrinsic bioactivity, limited cell adhesion without functionalization	Controlled drug delivery, injectable scaffolds with adjustable release profiles	[19,39,40,41,42]
Semi-synthetic hydrogels	GelMA, methacrylated hyaluronic acid (HAMA/AcHyA), PEG-fibrinogen	Combine natural bioactivity with tuneable crosslinking, controllable mechanical properties, and adjustable degradation	Require chemical modification steps; potential variability depending on functionalization	Injectable or photo-crosslinkable hydrogels widely used in BTE	[19,28,39,42]
Hybrid/composite hydrogels	GelMA-PEG, alginate-PEG, hydrogels reinforced with secondary delivery systems or bioactive ceramics (e.g., hydroxyapatite)	Balance biological functionality and mechanical stability; enhanced multifunctionality; improved structural integrity	Increased system complexity and potential challenges in degradation control	Advanced drug delivery platforms integrating cells, drugs, and bioactive particles	[43,44]

**Table 2:** Therapeutic cargoes delivered by hydrogels in cranial BTE. Summary of the main therapeutic cargo classes incorporated into hydrogel systems, including representative examples, biological functions, typical release strategies or profiles, and representative preclinical evidence. Where available, animal models, cranial defect characteristics, evaluation methods, and principal regenerative outcomes are reported, together with the main advantages, limitations, and relevant references.

Type of Cargo	Representative Examples	Main Biological Effects	Typical Release Strategy/Profile	Representative Preclinical Evidence and Main Outcomes	Main Advantages	Main Limitations	References
Growth factors	BMP-2, VEGF, FGF-2	Osteogenesis, angiogenesis, recruitment and differentiation of progenitor cells	Physical encapsulation, electrostatic or affinity interactions, covalent tethering, degradation-mediated release, and sequential delivery. These approaches generally prolong local retention, reduce burst release, and sustain biological signalling	Rat and mouse calvarial defect models. Sustained or sequential delivery of BMP-2, VEGF, and FGF-2 enhanced osteogenic marker expression, vascularisation, bone matrix deposition, bone volume, and defect filling	High biological potency, direct osteoinductive and pro-angiogenic activity	Short half-life in free form, dose-dependent adverse effects, uncontrolled diffusion, risk of ectopic mineralization	[59,60,61,62,63,64]
Peptides	RGD, LL-37, OGP, CPNE7-derived peptides	Cell adhesion; osteogenesis, immunomodulation, angiogenesis, antimicrobial activity	Covalent immobilization, physical encapsulation, self-assembly, or degradation-controlled release. Hydrogel incorporation protects peptides from enzymatic degradation and prolongs their local bioactivity	Rat calvarial defect models. Self-assembling peptide hydrogels increased bone volume, while RGD-functionalized multifunctional hydrogels promoted cell adhesion, M2 macrophage polarization, neovascularisation, and accelerated regeneration in a 5 mm critical-sized defect	High specificity, ECM-mimetic activity, adaptable biological functionality, lower structural complexity than growth factors	Rapid enzymatic degradation, short in vivo half-life, physicochemical instability, concentration-dependent activity	[65,66,67,68,69,70,71,72,73,74,75]
Small molecule drugs	Simvastatin, metformin, icariin, DEX, DFO	Osteogenesis, angiogenesis, immunomodulation, regulation of oxidative and inflammatory responses	Diffusion- or degradation-controlled release, frequently combined with NPs or composite hydrogels to improve retention and reduce rapid diffusion. Stimuli-responsive release may also be employed	Rat and rabbit cranial or craniofacial defect models. Sustained local delivery promoted osteogenic differentiation, vascularisation, M2 macrophage polarization, matrix deposition, and new bone formation compared with unloaded systems or free drug administration	Chemical stability, relatively low cost, low immunogenicity, easier synthesis and manufacture than recombinant proteins	Rapid diffusion and poor local retention when freely incorporated, dose-dependent or off-target effects, limited aqueous solubility for some compounds	[76,77,78,79,80,81,82,83,84,85,86,87,88,89]
Bioactive ions	Ca^2+^, Mg^2+^, Sr^2+^, Zn^2+^, Cu^2+^, Si^4+^	Osteogenesis, angiogenesis, immunomodulation	Sustained diffusion or degradation-coupled release from mineral phases, ceramics, NPs, chelated networks, or ion-containing composite hydrogels	Rat and mouse calvarial defect models. Controlled ion release enhanced osteogenic and angiogenic marker expression, macrophage polarization, vascular ingrowth, matrix mineralization, and new bone formation	High chemical stability, low cost, simultaneous structural and biological functions, potential mechanical reinforcement	Narrow therapeutic window, risk of local cytotoxicity or abnormal mineralization, release strongly dependent on material degradation and ion concentration	[33,48,90,91,92,93,94,95,96,97,98,99,100,101]
Nucleic acids	miRNA, siRNA, plasmid DNA, aptamers, tFNAs	Gene regulation, stem-cell recruitment, osteogenesis, angiogenesis, modulation of inhibitory pathways	Sustained or sequential release from protective hydrogel matrices, complexation with polyplexes, NPs, aptamers, or DNA nanostructures improves stability and cellular uptake	Rat and mouse critical-sized calvarial defect models. Hydrogel-mediated delivery enhanced endogenous cell recruitment, osteogenic gene expression, vascularisation, mineralization, bone volume, and defect repair	High molecular specificity, programmable regulation of regenerative pathways, prolonged local gene modulation	Enzymatic instability, inefficient cellular uptake; delivery complexity, possible off-target effects, manufacturing and regulatory challenges	[102,103,104,105,106,107,108,109,110,111,112,113,114,115]

**Table 4:** Secondary delivery systems integrated into hydrogels for cranial bone regeneration. Summary of the main secondary delivery systems integrated into hydrogels, including NPs, extracellular vesicles, microspheres, and stimuli-responsive components, highlighting their delivery functions, advantages and limitations.

Secondary System	Representative Examples	Main Delivery Function	Advantages	Main Limitations	Reference
Inorganic NPs	HAp, MSNs, bioactive glass, MOFs	Sustained release, osteoconduction	Mechanical reinforcement, ion release	Aggregation, complexity	[26,31,221,223,224,225,226,227,228,229]
Organic NPs	PLGA, chitosan NPs, lipid NPs	Sequential and controlled release	Biodegradable, versatile	Burst release, fabrication issues	[32,160,230,231,232,233]
Hybrid NPs	PLGA/Fe_3_O_4_, MSN/chitosan	Stimuli-responsive delivery	Multifunctionality	Increased complexity	[234,235]
EVs	MSC-derived EVs, engineered exosomes	Cell-free paracrine signalling	High bioactivity	Isolation and scalability	[236,237,238,239,240,241,242,243]
MPs	PLGA microspheres, gelatin microspheres, alginate microspheres	Long-term controlled release of growth factors and drugs	High loading capacity, prolonged release, protection of bioactive molecules	Larger size, possible heterogeneous distribution within hydrogels	[49,66,244,245,246,247,248,249,250,251]

## Data Availability

No new data were created or analyzed in this study. Data sharing is not applicable to this article.
